# Cholinergic modulation of hippocampal CA1 pyramidal cell excitability in *Arx*^GCG+7^ mice

**DOI:** 10.1016/j.expneurol.2025.115591

**Published:** 2025-12-11

**Authors:** Christopher M. Johnson, Joseph Dong, Peace Senkungu, Jahnvi Chandar, Natalia Ochoa-Sepulveda, Almedia J. Myers, Eric D. Marsh

**Affiliations:** a Division of Child Neurology and CHOP Research Institute, Children’s Hospital of Philadelphia, USA; b University of Pennsylvania Perelman School of Medicine, Philadelphia, PA, USA

**Keywords:** Developmental and epileptic encephalopathy, Infantile spasms syndrome, West syndrome, Aristaless related homeobox, Hippocampus, Acetylcholine, Depolarization block

## Abstract

Developmental and Epileptic Encephalopathies (DEE) are rare neurodevelopmental disorders defined by seizures, developmental delays, and abnormal EEG patterns. Infantile Spasms Syndrome (ISS), the most common DEE, was one of the first linked to single-gene variants, including polyalanine expansions in the *Aristaless Related Homeobox* (*ARX*) gene. The Arx^GCG+7^ mouse, which models the human mutation, exhibits behavioral phenotypes, epileptiform hippocampal activity, and reduced cholinergic input to the hippocampus. The role of the disrupted cholinergic signaling has not been explored in this model and may be contributing to hippocampal dysfunction and DEE pathogenesis. Hence, we investigated the impact of the *ARX* GCG expansion on the septo-hippocampal network by first reproducing the changes in Choline acetyltransferase (ChAT) protein levels in the medial septum (MS) and diagonal band of Broca (DBB). Next, we performed ex vivo patch-clamp recordings in CA1 pyramidal neurons and measured responses to cholinergic agonists. Finally, we assessed acetylcholine receptor expression by western blot across the hippocampus to assess for homeostatic changes in receptors. Our data demonstrated a ~ 60 % reduction in ChAT expression in the MS and DBB of Arx^GCG+7^ mice. The Arx^GCG+7^ CA1 pyramidal neurons exhibited increased firing activity and reduced sag potential. Despite reduced ChAT, carbachol- and pilocarpine-induced firing responses were preserved, while nicotinic receptor-mediated excitation was abolished. Carbachol-induced depolarization block threshold was elevated in mutants. Molecular analysis revealed increased muscarinic receptor 1 expression and decreased β4 nicotinic receptor expression in CA1.Together, these findings demonstrate histological, electrophysiological, and molecular alterations in the septo-hippocampal network of Arx^GCG+7^ mice. Together, they suggest impaired cholinergic signaling contributes to ISS pathogenesis and highlight the cholinergic system as a potential therapeutic target.

## 1. Introduction

Developmental and Epileptic Encephalopathies (DEEs) are syndromes whose common symptoms include seizures, intellectual impairment and sleep dysregulation amongst other neurological features (Scheffer et al., 2024). These conditions, which include named disorders such as Ohtahara Syndrome, Dravet Syndrome, and Infantile Spasms Syndrome (ISS) begin during infancy and affect approximately 1 in 2000 live births in total (Howell et al., 2018; Symonds et al., 2019). Though treatments are emerging for specific genetic conditions, better treatment options can emerge when the mechanisms causing these disorders are understood (FitzPatrick and Bird, 2022; Rahimi Darehbagh et al., 2024).

An increasing list of genes have been linked to the DEEs, which also can be caused by injuries to the developing brain. Understanding how local network changes (by gene dysfunction or injury) drive seizures and ID in these children are important for therapeutic development. One system that has not been studied in the DEEs has been changes in the septal-hippocampal cholinergic system. Acetylcholine (ACh), originating from the medial septum (MS) and diagonal band of broca (DBB) of the basal forebrain (Frotscher and Léránth, 1985), is a significant modulator of hippocampal and frontal cortical network activity and alterations in cholinergic function, through either receptor dysregulation or changes in production have been associated with epileptogenesis (Conti et al., 2015; Turski et al., 1983; Villa et al., 2019; Zerem et al., 2013), learning and memory disruptions (i.e. intellectual disabilities) and sleep disorders (Becchetti et al., 2020; Hasselmo, 2006).

At both network and cellular levels, cholinergic input to the hippocampus plays an important role in modulating several hippocampal functions. At the network level, it modulates prominent brain rhythms, gamma and theta frequency oscillations (Betterton et al., 2017; Kramis et al., 1975). On the cellular level, ACh has diverse effects, increasing or inhibiting synaptic activity depending on the presynaptic location of different AChR subtypes (Goswamee and McQuiston, 2019; McQuiston, 2014). Furthermore, ACh also increases pyramidal cell excitability (Benardo and Prince, 1982a) and lowers the threshold for depolarization block (DB) (Fraser and MacVicar, 1996).

In addition to playing a significant role in modulating cellular and network activity, cholinergic signaling plays an important role in neuronal development as well. Activation of α7-nicotine acetylcholine receptors (nAChRs) promotes glutamatergic synapse formation and long-term potentiation in the hippocampus (Freund et al., 2016; Lozada et al., 2012). In addition, nicotine can induce plasticity (Collo et al., 2013) and ACh promotes neurogenesis (Jeong et al., 2014; Tisch et al., 2014). Thus, disruptions in MS and DBB neurons could be complicit in the neurological symptoms of DEEs.

The most common DEE, ISS, can be caused by mutations in the aristaless-related homeobox gene (*ARX*) located on the X chromosome. Pathogenic variants in *ARX* have also been associated with brain malformations, Intellectual Disabilities, Autism and other neurological features (Bienvenu, 2002; Guerrini et al., 2007; Kato et al., 2004; Strømme et al., 2002). Mutations in *ARX* primarily affect males due to the X-linked recessive inheritance pattern. *ARX* encodes for a transcription factor, which acts as both a repressor and activator (Seufert et al., 2005). The most common mutations, comprising approximately 60 % of cases, are polyalanine expansions caused by 7 GCG repeats in the first and second alanine tracts (Shoubridge et al., 2010) and believed to cause a loss of function (Nasrallah et al., 2004). Mouse models containing polyalanine expansions in *Arx* (*Arx*^GCG+7^), hereafter referred to as GCG mice, recapitulate several symptoms seen in human patients such as seizures, motor impairment, and cognitive dysfunction (Jackson et al., 2017; Kitamura et al., 2009).

Studies from our lab and others indicate that the hippocampal network of *Arx* mutant mice can generate seizures and displays abnormal activity, possibly accounting for some of their behavioral impairments (Beguin et al., 2013; Joseph et al., 2021). Furthermore, Kitamura et al. (2009) demonstrated that cholinergic neurons in the basal forebrain are drastically decreased by ~77 % in *GCG* mice (Kitamura et al., 2009), but how disruption of the cholinergic system impacts hippocampal function and contributes to ISS pathogenesis is unknown. Thus, we investigated the role of septo-hippocampal dysfunction in *Arx*-induced epileptogenesis and cognitive impairment using an *GCG* mouse model to provide a framework for novel treatment avenues that could be found more broadly in other DEEs. Our findings support a homeostatic cholinergic compensation model, in which receptor-level remodeling and impaired inhibitory mechanisms may contribute to network dysfunction.

## 2. Materials and methods

### 2.1. Animals

*Arx*^GCG+7^ mice were generated by crossbreeding female *Arx*^GCG+7/X^ mice with wild-type (WT) male mice on a C67Bl/6 background. The offspring were genotyped using the following primers: GCG Fwd: AA GGC GAA AAG GAC GAG GAA AGG, GCG rev: CTT TAG CTC CCC TTC CTG GCA CAC, Kita Neo: TGT TCA ATG GCC GAT CCC AT. Only male mice ≥p30 were used for experiments. Male mice were used exclusively, as the disorder occurs predominantly in males. The *Arx*^GCG+7/X^ mice were provided by the RIKEN BRC through the National BioResource Project of the MEXT, Japan. All animals were treated in accordance with the National Institutes of Health Guide for the Care and Use of Laboratory Animals and approved by the Children’s Hospital of Philadelphia Institutional Animal Care and Use Committee.

### 2.2. Immunofluorescence

Mice p30-p40 were anesthetized with isoflurane and transcardially perfused with 3 % PFA, 1 % Picric Acid, and 0.1 % glutaraldehyde in 1× phosphate-buffered saline (PBS). The brain tissue from each mouse was dissected and post fixed overnight in the same fixative. After post-fixation, the samples were placed in 10 % sucrose in PBS until the brains sank (~3 h), then 20 % sucrose and, 30 % sucrose in 1× PBS overnight until the tissue was fully submerged. The samples were embedded and frozen in optimum cutting temperature (OCT) compound and stored at −80 °C until use. The brain tissue samples were then serially sectioned, coronally, using a cryostat at a thickness of 20 μm.

ChAT staining: The slides were incubated in the Mouse On Mouse (M. O.M.) IgG kit (VWR; Cat. No.: 101097–890) for 1 h at room temperature followed by blocking with 20 % normal rabbit serum, 5 % bovine serum albumin (BSA), 0.2 % fish gelatin, 0.5 % Triton X-100, and 1× PBS for 1 h at room temperature. Sections were incubated in an anti-ChAT antibody (1:100; Millipore Sigma; Cat. No.: AB1582) diluted in blocking solution for 36 h at 4 °C. Following primary incubation, sections were washed three times in 1× PBS and incubated with AlexaFluor 488 secondary antibody (1:40; ThermoFisher Scientific; Cat. No.: A11055) in blocking solution for 1.5 h at room temperature (20 °C). Tissue sections were counterstained with DAPI (1:1000; Invitrogen; Cat. No.: D1306), cover slipped with 2–3 drops of Fluoromount-G (Southern Biotech; Cat. No.: 0100–01) and stored at 4 °C before imaging.

NeuN staining: Sections were blocked in 5 % normal donkey serum (NDS), 5 % normal goat serum (NGS), 1 % bovine serum albumin (BSA), and 0.2 % fish gelatin in 1× PBS for 1 h at room temperature. Tissue was then incubated in an anti-NeuN primary antibody (1:100; Millipore; Cat. No.: MAB377) diluted in blocking solution overnight at 4 °C. Following primary incubation, sections were washed three times in 1× PBS and incubated with AlexaFluor 594 donkey anti-goat secondary antibody (1:200; Invitrogen; Cat. No.: A11058) diluted in blocking solution for 30 min at room temperature (20 °C). Sections were counterstained with DAPI (1:1000; Invitrogen; Cat. No.: D1306), cover slipped with 2–3 drops of Fluoromount-G (Southern Biotech; Cat. No.: 0100–01) and stored at 4 °C before imaging.

### 2.3. Image analysis

Sections were imaged using a Leica DMi8 inverted microscope equipped with a Leica DFC9000 GT camera. Tiled images taken with a 20×/0.4 NA objective were stitched together using Leica Application Suite X software. Higher magnification images were taken with a 40×/0.95 NA objective. Fluorescence excitation was provided by a Leica LED light source, using the following excitation filter sets: 405/60 nm (model 38,549), 470/40 nm (model 55,254), and 545/25 nm (model 256). Images, all acquired at 20× magnification, were captured in batches during simultaneous imaging sessions. Similar microscope settings, including gain, offset, and exposure time, were used across all groups. Post-acquisition image processing was limited to brightness and contrast adjustments applied equally to all images. ChAT+ cells were counted manually, while NeuN+ cells were quantified using an automated method in Fiji (Schindelin et al., 2012). Data were quantified by counting the number of positive cells in a consistently sized defined region and divided by the total area. Six sections from five mice for each genotype were analyzed, including two sections from a single mouse to maximize the use of high-quality tissue. Statistical analyses were conducted using individual sections as the unit of analysis.

### 2.4. Western blot

Microdissection: Brains from mice aged p30–p45 were quickly removed and put in ice-cold sucrose artificial cerebrospinal fluid (ACSF). Coronal sections containing the hippocampus were cut at 600 μm using a Leica Vibratome VT12000S and placed in ACSF. The hippocampus was isolated from surrounding tissue and separated by cutting along dentate gyrus (DG) mossy fibers and making an oblique cut along the CA3 to dissect the CA1, CA3, and DG. The tissue was immediately frozen using dry ice and stored at −80 °C.

Protein Preparation: Tissue samples were homogenized in RIPA buffer containing protease inhibitors using a sonicator. Lysates were centrifuged at 14,000 rpm at 4 °C and the resulting supernatants were frozen at −80 °C. Protein concentration was determined using a Bicinchoninic Acid (BCA) assay.

Western Blot: Protein samples were loaded into 10 % precast gels from Invitrogen and run at 120 V for approximately one hour. Proteins were transferred to polyvinylidene difluoride (PVDF) membranes using wet transfer. Membranes were blocked in 5 % bovine serum albumin (BSA) for one hour and incubated in the antibody at 4 °C overnight. The following primary antibodies were used for Western blot analysis: Antibodies targeting muscarinic receptor (mAChR) subunits included CHRM1 (rabbit polyclonal; Bioss; Cat. No.: BS-1150R; 1:200), CHRM2 (rabbit polyclonal; Thermo Fisher Scientific; Cat. No.: BS-0441R; 1:300–1:1000), and CHRM4 (rabbit polyclonal; Invitrogen; Cat. No.: PA5–77483; 1:200). Antibodies targeting nAChR alpha subunits included CHRNA3 (rat monoclonal; Santa Cruz Biotechnology; Cat. No.: sc-58,605; 1:1000), CHRNA4 (rabbit polyclonal; Thermo Fisher Scientific; Cat. No.: BS-1038R; 1:200), and CHRNA7 (mouse monoclonal; Millipore; Cat. No.: MABN529; 1:1000). Beta subunit antibodies included CHRNB2 (rabbit polyclonal; Bioss; Cat. No.: BS-4247R; 1:500) and CHRNB4 (rabbit polyclonal; Novus Biologicals; Cat. No.: NBP2–60788; 1:1000).

Following primary antibody incubation, membranes were washed in Tri-Buffered Saline with 0.1 % tween (TBST) the next day and incubated with the appropriate secondary antibodies: IRDye 680RD (LI-COR Biosciences; P/Ns: 926–68,070 and 926–98,073) and IRDye 800CW (LI-COR Biosciences; P/Ns: 926–32,210 and 926–32,211. Total protein stains were performed using Revert^™^ 700 Total Protein Stain. Blots were imaged using the LI-COR Odyssey imaging system.

Blot quality was determined by a trained evaluator blinded to the experimental conditions. Criteria included uniform protein transfer without bubbles or streaks, absence of membrane tears or folds, clear and consistent total protein staining across the membrane, and absence of background artifacts. Only blots meeting these quality standards were included in the analysis. Although GAPDH was probed, normalization was performed using total protein staining, with regions selected for quantification free of artifacts. Antibody specificity was validated through several measures. Detection of a single band at the expected molecular weight was confirmed using protein lysates from control whole brain tissue. The linear range of detection was established by serial dilution of protein lysates, and signal intensity was proportional to protein concentration within the range used for all subsequent experiments.

### 2.5. Electrophysiology

Mice were anesthetized with isoflurane and transcardially perfused with ice-cold sucrose-based ACSF containing (in mM) 192 sucrose, 2.5 KCl, 0.5 CaCl_2_, 10 MgSO_4_, 26 NaHCO_3_, 1.25 NaH_2_PO_4_, 12.2 d-glucose, 3 sodium pyruvate, 5 sodium ascorbate and 2 thiourea. Mice were decapitated and the brain was rapidly placed in ice-cold sucrose-based ACSF. The solution was bubbled with 95 % O_2_ balanced with 5 % CO_2_ (pH 7. 40). Brains were mounted dorsally on an agar block at a 10–15° angle. Transverse slices at 350 μm were cut from mice p30-p45. Slices were transferred to recovery solution ACSF containing (in mM) 115 NaCl, 2.5 KCl, 1 MgSO_4_, 2.5 CaCl_2_, 1.4 NaH_2_PO_4_, 24 NaHCO_3_, 12.5 glucose, 5 sodium ascorbate, 3 sodium pyruvate, and 2 thiourea for 45 min at 32 °C and room temperature each. After recovery, slices were transferred to a recording chamber containing recording ACSF containing (in mM) 128 NaCl, 2.5 KCl, 1 MgSO_4_, 2.5 CaCl_2_, 1.4 NaH_2_PO_4_, 26 NaHCO_3_, 12.2 glucose. Slices were perfused with carbogenated ACSF at a rate of approximately 2 ml/min using a gravity-based system and maintained at 32 °C while recording.

Whole cell current clamp recordings were performed on slices containing the hippocampus visualized on a screen via a charge-coupled device (CCD) camera system to an Olympus BX51WI microscope. Cells were selected based on location and size. Recordings were discarded if the cell had a resting membrane potential greater than −55 mV, action potential amplitude less than 60 mV prior to drug exposure, or if access resistance increased by >20 % during the recording. Agonists used were Carbamylcholine Chloride (Sigma; Cat. No.: C4382–1G), Nicotine hydrogen tartrate salt (Sigma; Cat. No.: N5260–25G), and Pilocarpine hydrochloride (Tocris; Batch #: 2 A/280529). Slices were discarded after one drug exposure. Patch pipettes with a resistance of 3–6 MΩ were pulled from borosilicate glass with a Sutter pipette puller (P-97). Pipettes were filled with solution containing the following (in mM) 135 potassium-gluconate, 5 KCl, 2 NaCl, 10 HEPES, 4 Mg-ATP, 0.3 Na-GTP, and 4 EGTA (pH 7.20). The liquid junction potential was estimated to be ~13 mV and was not corrected. Signals were amplified with a Multi-clamp 700B amplifier (Molecular Devices, Union City, CA), sampled at 10 kHz, filtered at 2 kHz using the low-pass filter, digitized using a Digidata 1440 A and collected with the Clampex 10.3 data acquisition software.

Resting membrane potential was determined based on recordings with membrane potentials without current injection. Input resistance was measured from the slope of the linear portion of the current-voltage response to hyperpolarizing current injections. The inward rectification ratio was calculated as the input resistance measured before the onset of the sag potential, typically occurring early in the current-voltage response to hyperpolarizing current injections, divided by the input resistance during the sag potential at approximately −100 mV. Rheobase was defined as the minimum amount of current needed to evoke action potentials in 20 pA steps. Membrane time constant was calculated as a single exponential function fitted to an early hyperpolarizing current step. Sag potential was calculated as the difference between steady state potential at −100 mV and peak potential. Depolarization block was defined as a failure to generate action potentials after a minimum of 75 % of the duration of the current injection, characterized by either a steady membrane potential or membrane depolarizations that did not exceed 20 mV. All determinations were made by a trained human rater through visual inspection of the membrane voltage trace.

Action potential properties were measured from the first non-bursting action potential at rheobase. AP threshold was based on the derivative of the voltage (dV/dt). AP amplitude was measured from threshold to peak of first spike at rheobase. AP rise slope, decay slope, rise time, decay time, and half-width were measured from 10 % to 90 % of AP amplitude. AP half-width was determined based on the width of the AP at half amplitude. Fast afterhyperpolarization (fAHP) and medium afterhyperpolarization (mAHP) were measured from threshold to the respective lowest peaks. Spike frequency adaptation was calculated by generating the ratio of the peak frequency to steady state frequency.

Mice aged p30–p45 were used for all electrophysiological experiments and all experimental groups were age-matched. Baseline data from all experimental groups was aggregated, as the baseline measurements were collected under identical conditions and did not differ significantly across experiments.

### 2.6. Statistics and data analysis

All data sets were first tested for normality using a Shapiro-Wilk test. Data that were found to be normally distributed were analyzed using parametric tests, while non-normally distributed data were analyzed with appropriate non-parametric alternatives. For immunofluorescence and western staining data, students t-test was applied with corrections for multiple comparisons. Western blot results were quantified using Image J. The electrophysiological data were analyzed with Clampfit 10.3 software (Molecular Devices). Statistical analysis was performed in GraphPad Prism 10. For electrophysiology, a two-way repeated measures ANOVA (genotype and treatment) for evoked firing frequency data with Tukey’s post hoc and Mixed Effects Model with Fisher’s LSD were used for all pairwise comparisons. For baseline frequency data, pairwise *t*-tests were conducted at individual current steps to explore genotype differences in firing rate. To control for multiple comparisons, the two-stage step-up procedure of Benjamini, Krieger, and Yekutieli (BKY) was used. This ensured the detection of specific group differences that may not be identified by the omnibus ANOVA alone (Midway et al., 2020). Data are presented as means ± SE. Differences were considered significant when *P* ≤ 0.05.

## 3. Results

### 3.1. ChAT expression in the basal forebrain is decreased

**Fig. 1.**
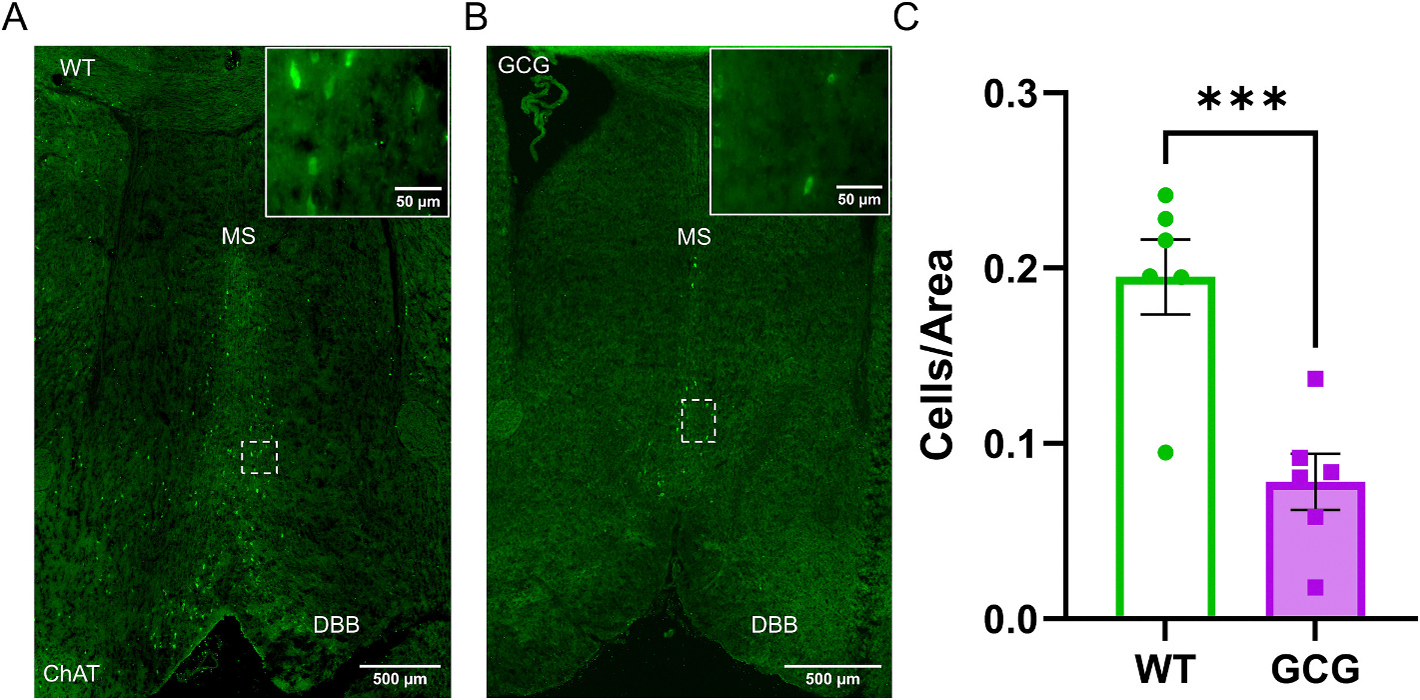
ChAT expression is decreased in the basal forebrain of *Arx*^GCG+7^ mice. (A, B) Immunofluorescence images of ChAT expression in the medial septum (MS) and Diagonal Band of Broca (DBB) of wild-type (WT) and *Arx*^GCG+7^ (GCG) mice. Dashed boxes indicate regions shown at higher magnification (40×) in the insets. (C) Quantification of ChAT+ cells in the MS and DBB, normalized to area. Data are presented as mean ± SEM (WT, n=6 sections/5 mice; GCG, n=6 sections/5 mice). Statistical significance was determined using a one-tailed unpaired t-test. *P < 0.05, **P < 0.01, ***P < 0.001.

Kitamura et al. (2009) documented significant loss of cholinergic input from the basal forebrain to the hippocampus. Prior to studying the electrophysiological alterations from this decrease, we wanted to reproduce these experiments. We quantified the population of ChAT+ cells in the basal forebrain of both GCG and WT mice by immunofluorescence for ChAT expression and found a notable reduction of ChAT density in GCG mice compared to WT littermates (WT, 0.20 ± 0.5 cells/μm^2^, *n* = 6 sections/5 mice; GCG, 0.08 ± 0.04 cells/μm^2^, n = 6 sections/5 mice; *p* = 0.001; Fig. 1), similar to previous reports (Kitamura et al., 2009).

Though ChAT expression is decreased in the MS and DBB, there is little to no cell death reported in GCG mice (Price et al., 2009; Siehr et al., 2020). One explanation for this discrepancy is that the cholinergic cells precursors in the MS and DBB have changed their cell fate such that the cell bodies are still present. Thus, we stained for NeuN within the MS and DBB to determine if total neuron count is decreased. NeuN expression was not significantly altered (WT, 14.4 ± 8 cells/μm^2^, n = 6 sections/3 mice; GCG, 18.2 ± 13.8 cells/μm^2^, n = 6 sections/3 mice; *p* = 0.6; Supp. Fig. 1). These results suggest that the GCG mutation disrupts ChAT expression in the basal forebrain without altering overall number of NeuN-positive cells. Consequently, the reduction in ChAT expression may lead to decreased ACh synthesis and, consequently, reduced ACh release, which may impair hippocampal physiology.

### 3.2. CA1 pyramidal cell excitability is increased in GCG mice

GCG mice develop seizures and exhibit electroencephalographic seizure activity in multiple brain areas, especially within the hippocampus (Kitamura et al., 2009; Price et al., 2009), suggesting altered excitability of various neuronal populations. Changes in postsynaptic intrinsic excitability and altered cholinergic input can both contribute to the epileptic state found in these mice. Thus, we first measured CA1 pyramidal cells (CA1Ps) intrinsic excitability by whole-cell current clamp recordings. We measured intrinsic membrane properties and action potential (AP) generation of CA1Ps in WT and GCG mice aged p30–p45 under baseline (BL) conditions. P30–45 ages were chosen to correspond a period of marked increase in seizure frequency in the model, as observed in both our unpublished data and in Jackson et al. (2017). This period aligns with the functional maturation of hippocampal circuits, including CA1Ps. Synaptogenesis peaks by ~P28 in rats (Steward and Falk, 1991), LTP reliability increases through P40 in mice (Ostrovskaya et al., 2020), and CA1P intrinsic properties stabilize by this stage (Dougherty and Kelly Dougherty, 2020), providing a consistent backdrop for assessing seizure-related changes. Further, *ARX* expansion patients typically survive into adulthood and continue to experience symptoms making this age clinically relevant.

**Fig. 2.**
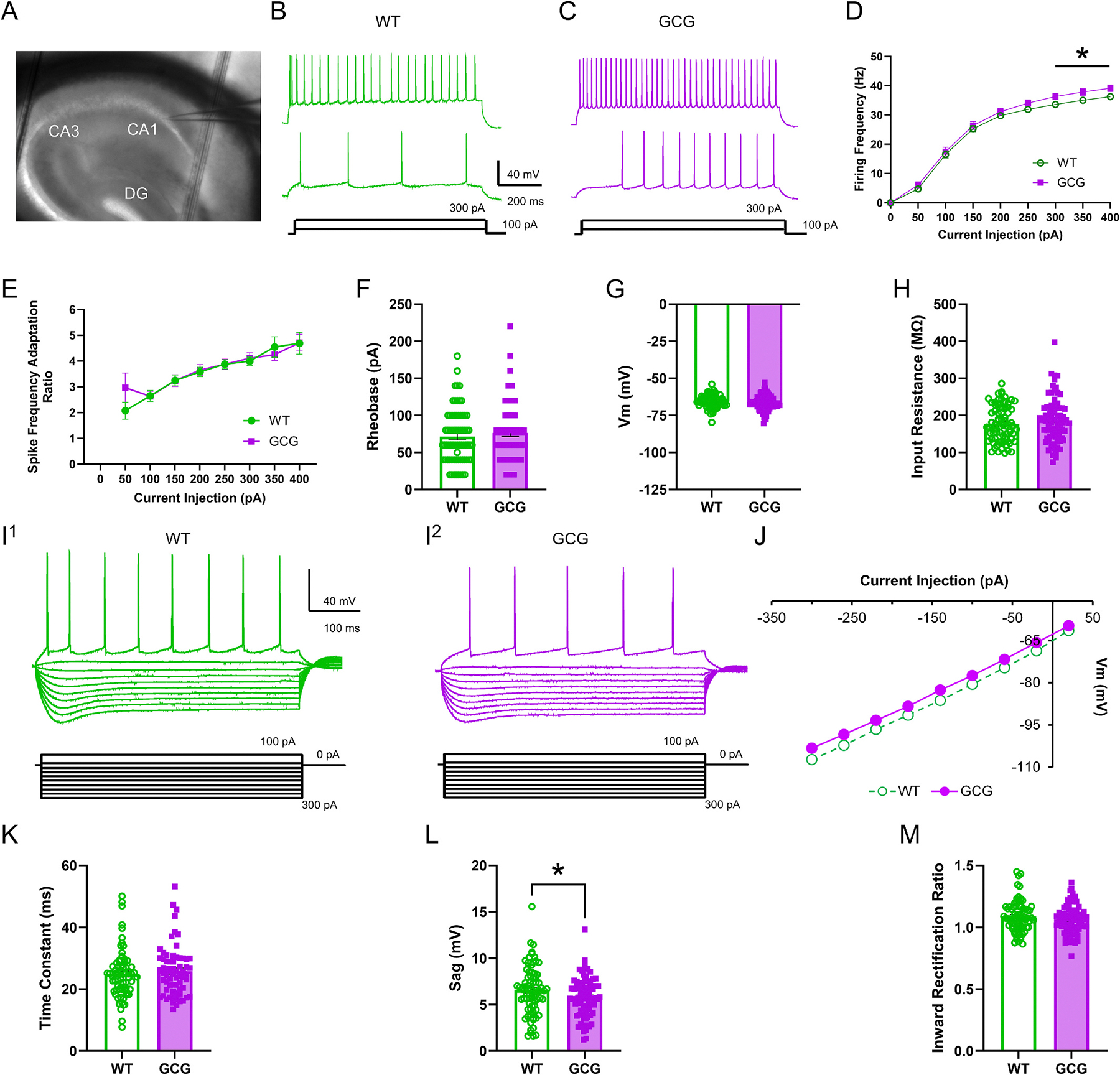
CA1 pyramidal cells are hyperexcitable in *Arx*^GCG+7^ Mice. (A) Representative image of a brain slice containing the hippocampus (B, C) Representative voltage responses from CA1 pyramidal cells (CA1Ps) in wild-type (WT) and GCG mice in response to 50 pA and 200 pA depolarizing current injections (1 s duration). (D) Quantification of spike frequency (E) and spike frequency adaptation in CA1Ps from WT and GCG mice in response to 50 pA current injections. (F-H) Quantification of rheobase (F), resting membrane potential (G), and (H) input resistance. (I^1^, I^2^) Whole-cell current-clamp recordings of CA1Ps from WT and GCG mice in response to hyperpolarizing current steps. (J) Representative I–V plots of CA1Ps. (K-M) Quantification of time constant (K), sag potential (L), and inward rectification ratio (M). Data are presented as mean ± SEM (WT, n=76 cells/42 mice; GCG, n=77 cells/37 mice). Statistical significance was determined using a two-way repeated-measures ANOVA with Tukey’s post-hoc test for firing frequency, action potential numbers, and spike frequency adaptation. Pairwise t-tests were conducted post hoc to explore group differences at individual current steps following a non-significant omnibus ANOVA. A Student’s unpaired t-test or Mann-Whitney U test were used for all other comparisons based on normality. *P < 0.05, **P < 0.01, ***P < 0.001.

First, action potential excitability was assessed by applying 50 pA depolarizing current injection steps to CA1Ps. In both WT and GCG cells, increasing depolarizing current injections reliably evoked increased AP firing activity (Current Injection, F(2.945, 444.6) = 1068, *p* < 0.0001; Fig. 2B,C). Initial analysis, did not reveal a significant main effect of genotype or interaction (Main effect of genotype, F_(1, 151)_ = 1.991, *p* = 0.1603; interaction of current injection × genotype, F_(8, 1208)_ = 1.380, *p* = 0.2009). However, Tukey’s post hoc comparisons revealed significantly increased firing activity at current injections of 300–400 pA in GCG cells compared to WT cells (Tukey’s post hoc analysis, *p* < 0.05; Fig. 2D,E). Though there was no significant overall group difference between genotypes, we conducted additional exploratory pairwise *t*-tests at individual current steps to further evaluate the apparent divergence in firing rates at higher input levels. To evaluate these observations, we conducted pairwise t-tests at individual current steps, applying correction for multiple comparisons to control for false positives. This analysis confirmed significant differences at current injections 300 pA – 400 pA (q = 0.048).

**Fig. 3.**
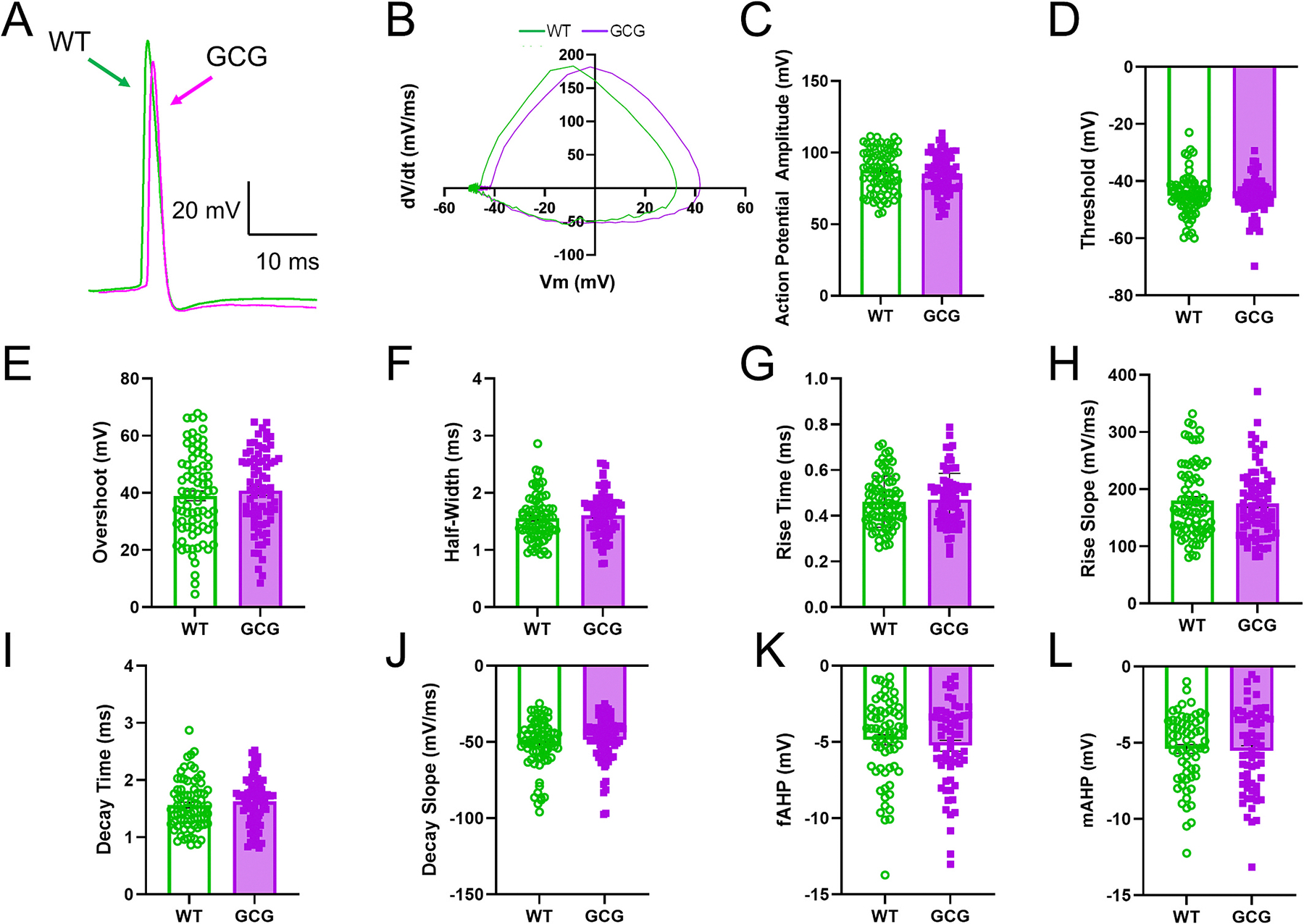
Action potential properties are unchanged in GCG mice. (A) Representative CA1P action potential morphologies from WT and GCG mice. (B) Representative phase plots from WT and GCG CA1Ps. (C-L). Quantifications of (C) action potential amplitude, (D) threshold, (E) overshoot, (F) action potential duration at half amplitude (G) rise time, (H) rise slope, (I) decay time, (J) decay slope, (K) fast afterhyperpolarization amplitude (fAHP), and (L) medium afterhyperpolarization amplitude (mAHP). Statistical significance was determined using an unpaired t-test or Mann-Whitney U test based on normality. *P < 0.05, **P < 0.01, ***P < 0.001.

Active and passive membrane properties were also assessed. There were no significant differences between the genotypes in rheobase, resting membrane potential, input resistance, membrane time constant, or inward rectification (Fig. 2G–M). However, the voltage sag potential of GCG cells was significantly smaller than the sag potential in WT mice (WT, 6.6 ± 2.6 mV, *n* = 76 cells/42 mice; GCG, 5.8 ± 2.1 mV, 77 cells/37 mice; *p* = 0.045; Fig. 2L). In addition, action potential (AP) properties were compared. AP amplitude, threshold, half-width, rise time, decay time, rise slope, decay slope overshoot, fAHP, and mAHP were assessed though there were no significant differences (Fig. 3). These findings indicate that CA1Ps in GCG mice have a modest increase in evoked firing relative to WT. As Arx is not expressed in CA1Ps at P30, the intrinsic excitability could be due to early developmental influences, a disrupted network, or altered cholinergic input into CA1.

### 3.3. Cholinergic modulation of CA1 pyramidal cells is preserved

Homeostatic responses are a common feature of neurons to altered synaptic input. We hypothesized that CA1Ps would increase their sensitivity to available ACh as a compensatory mechanism in response to the decreased cholinergic input. Furthermore, we hypothesized that this homeostatic response was likely through altered postsynaptic cholinergic receptor expression. Thus, alteration of cholinergic receptor expression in the hippocampus would likely lead to a significant change in electrophysiological response to cholinergic receptor agonism and potentially, aberrant network activity. To test this, we measured the firing activity and other membrane properties of CA1Ps in response to Carbachol (CCh) a well-known cholinergic agonist.

**Fig. 4.**
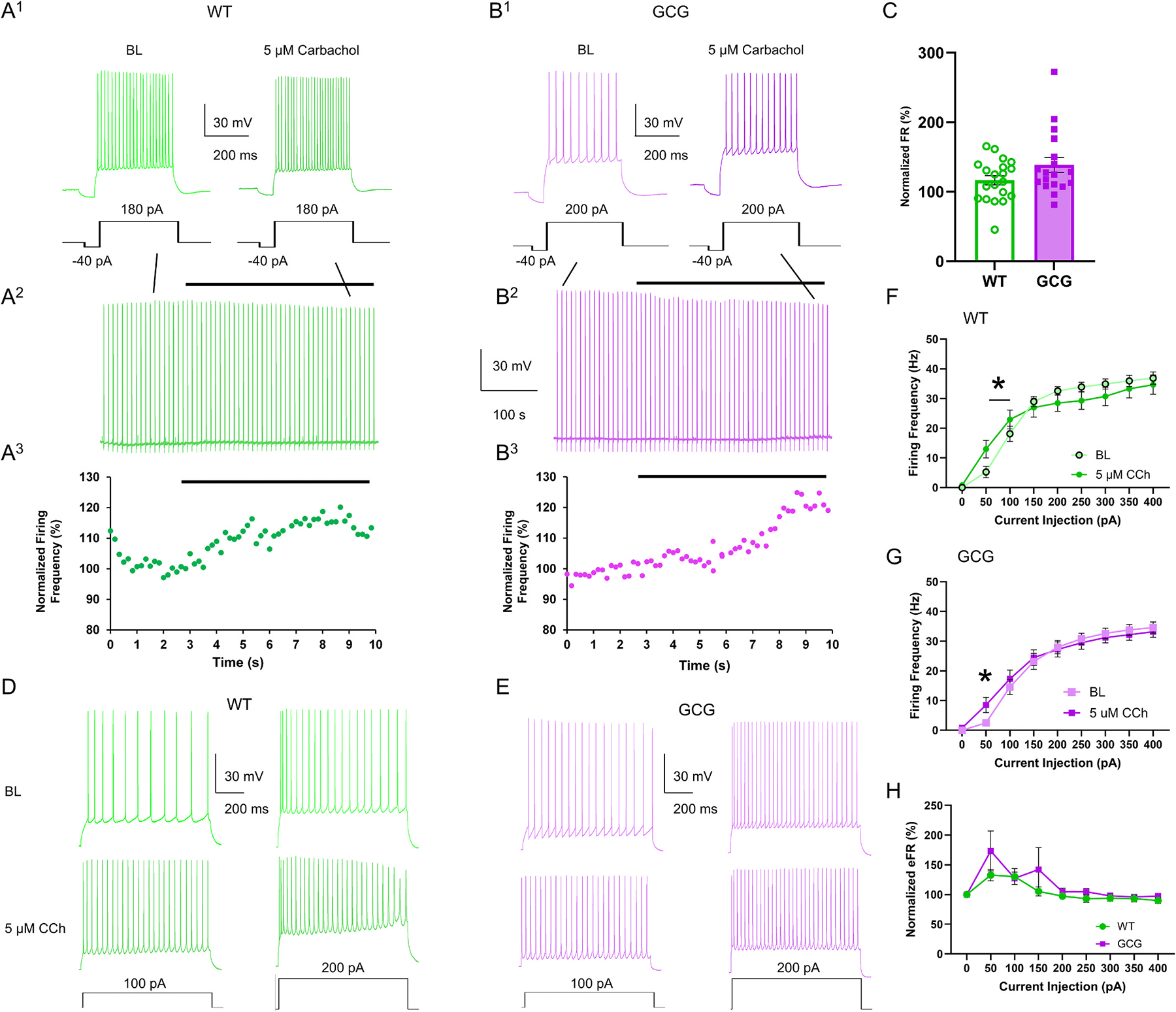
Carbachol enhances firing activity in CA1 pyramidal cells (CA1Ps) similarly in WT and GCG mice. (A^1^, B^1^) Representative firing responses of CA1Ps from wild-type (WT) and GCG mice, respectively, to current injections at baseline and during bath application of 5 μM carbachol. (A^2^, B^2^) Full trace recordings of CA1P responses to periodic current injections from WT and GCG cells, respectively. Bars indicate duration of 5 μM carbachol exposure. (A^3^, B^3^) Firing rates of CA1Ps normalized to the last minute of baseline activity. (C) Normalized quantification of firing rate. (D, E) Representative traces of evoked firing activity in CA1Ps from WT and GCG mice before and during bath application of 5 μM carbachol. (F, G) Quantification of firing frequency in response to 50 pA depolarizing current steps in WT and GCG mice, respectively. (H) Firing frequency normalized to baseline in response to carbachol administration. Data are presented as mean ± SEM (WT, n=22 cells/18 mice; GCG, n=21 cells/15 mice). Statistical significance was determined using a mixed effects model for WT data and two-way repeated-measures ANOVA with Tukey’s post-hoc test for GCG data. *P < 0.05, **P < 0.01, ***P < 0.001. BL, baseline, CCh, carbachol.

We bath applied 5 μM and 20 μM CCh and found dose related increases, so we present primarily the 5 μM data. We periodically gave 500 ms stimulating pulses that were 1.5–2× rheobase once every 10 s to evoke firing activity. With bath administration of CCh (5 μM), firing activity was significantly increased in both genotypes with no significant differences between genotypes (WT, 116.4 ± 6.7, GCG, 138.6 ± 10.9, *p* = 0.09; Fig. 4A–C). In each genotype, the AHP after the train of spikes was significantly decreased in the presence of CCh compared to baseline values (Main effect of drug, F_(1,31)_ = 20.59, *p* <0.0001; Supp. Fig. 2). We also recorded firing activity in response to 50 pA steps of depolarizing current injections up to 700 pA. In the presence of bath applied 5 μM CCh, evoked firing activity at lower current injections was significantly increased for both WT and GCG cells at 50 pA and 100 pA for WT and 50 pA for GCG (WT, Significant Interaction Current Injection × Drug, F_(8, 332)_ = 4.054, *p* = 0.0001; GCG, Main effect of drug, F(40, 320) = 14.50, *p* < 0.0001; Fig. 4D–G). However, CCh did not differentially affect the firing activity between genotypes (Main effect of genotype F_(1, 41)_ = 2.686, *p* = 0.11; Fig. 4H). Similarly, CCh significantly reduced spike frequency adaptation when normalized to BL to the same extent in both genotypes (Main effect of genotype F_(1, 41)_ = 2.973, *p* = 0.09; Supp. Fig. 3).

Though there were trends towards changes in rheobase, resting membrane potential, input resistance, and inward rectification after bath applying 5 μM CCh, only sag potential was significantly decreased (WT, BL 6.9 ± 3.7 mV; 5 μM CCh, 5.8 ± 3.0 mV; *n* = 22 cells/18 mice; *p* = 0.02. GCG, BL 6.1 ± 2.2 mV; 5 μM CCh, 5.1 ± 2.9 mV; *n* = 21 cells/15 mice; *p* = 0.02; Table 1). When comparing action potential properties, we found that amplitude, overshoot, rise slope, and decay slope were all significantly decreased by CCh while mAHP was significantly increased. Although CCh did not produce a differential effect between genotypes on these properties, half-width and decay time at baseline were found to be significantly different between genotypes (Table 1).

Similar increases in firing activity and reductions of spike frequency adaptation observed during 5 μM CCh administration were also seen when exposed to a higher concentration of CCh (20 μM) (Supp. Fig. 4A–D). Unlike 5 μM CCh, though, 20 μM CCh significantly decreased rheobase in both genotypes, increased input resistance and depolarized resting membrane potential only in the WT cells, which are consistent with previous findings (Supp. Fig. 4). Altogether, these findings demonstrate a preserved and dose dependent effect of CCh on CA1P excitability in GCG CA1Ps.

### 3.4. Carbachol-induced depolarization block threshold is increased

**Fig. 5.**
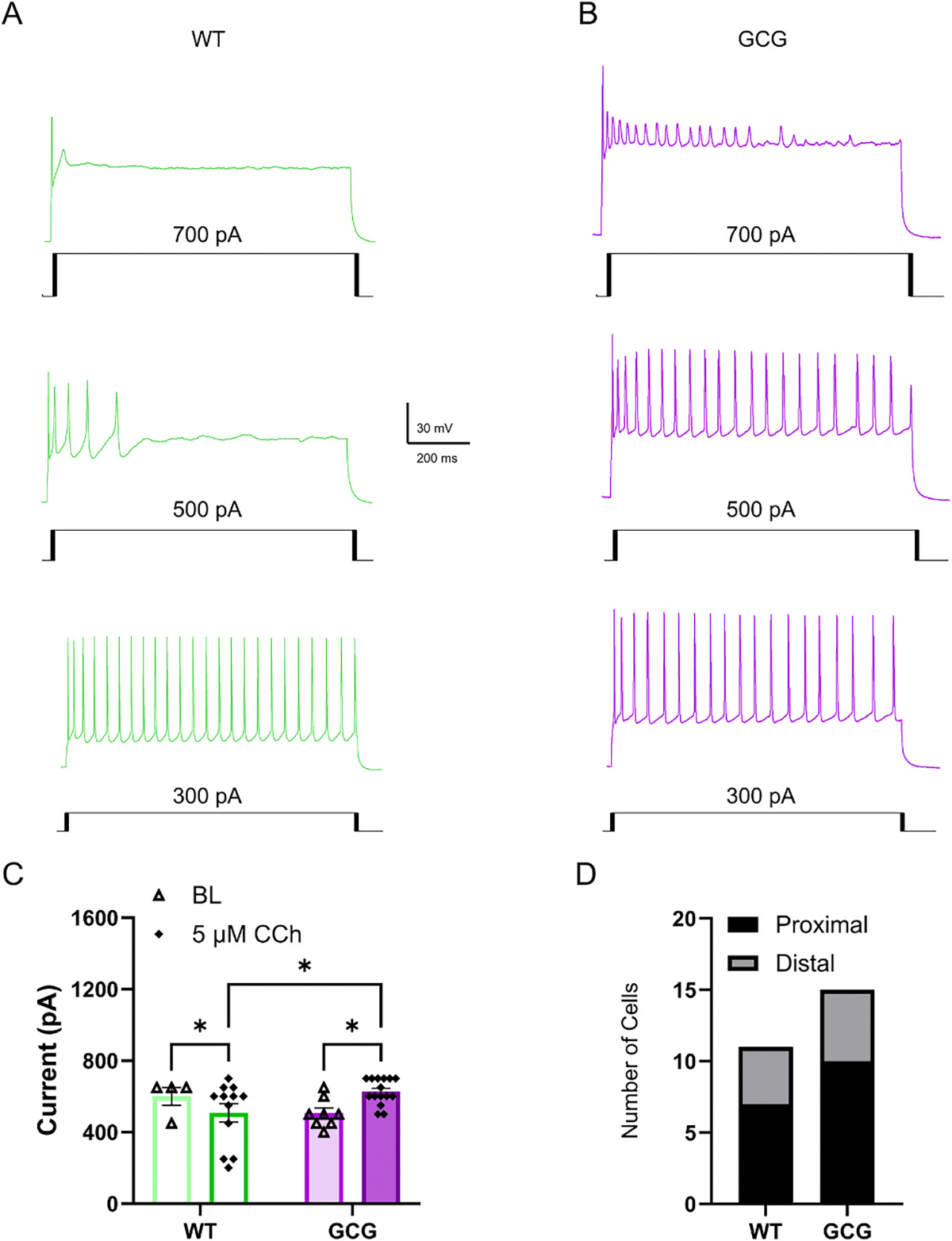
Carbachol increases the depolarization block threshold in GCG mice. (A, B) Representative traces showing depolarization block in CA1 pyramidal cells (CA1Ps) from wild-type (WT) and GCG mice during bath application of 5 μM carbachol (CCh). (C) Histogram of current thresholds for depolarization block in WT and GCG mice at baseline and during CCh exposure. (D) Proportions of CA1Ps exhibiting depolarization block in WT and GCG mice at proximal and distal hippocampal locations. Data are presented as mean ± SEM (WT, n=12 cells/11 mice; GCG, n=15 cells/12 mice). Statistical significance of depolarization block current thresholds was determined using a mixed-effects model with Fisher’s LSD post hoc analysis. Statistical significance of cell proportions was determined using chi-square analysis. *P < 0.05, **P < 0.01, ***P < 0.001. BL, baseline, CCh, carbachol.

DB is a characteristic of neurons that limits their firing capability in response to stimulation (Bianchi et al., 2012) and is also believed to play a crucial role in seizure dynamics. DB in CA1Ps, specifically, can be induced by enhanced cholinergic input whose disruption may increase susceptibility to seizures (Knauer and Yoshida, 2019). Therefore, we assessed the current at which DB occurred in CA1Ps up to 700 pA current injection before and during bath application of 5 μM CCh. At baseline, DB was observed in 4 out of 22 WT cells at an average current of 600 pA and in 8 out of 21 GCG cells at an average current of 506 pA, which were not significantly different (Significant Interaction Genotype × Drug, F_(1,10)_ = 13.64, *p* = 0.004, Uncorrected Fisher’s LSD, *p* = 0.06; Fig. 5 A–C). During CCh exposure, however, the number of cells that went into DB increased in both genotypes (WT, *n* = 12; GCG, *n* = 15) and while the current at which DB occurred in WT cells decreased, average DB current in GCG cells increased, which was significantly different from WT (WT, 508 ± 177 pA, n = 12 cells/11 mice; GCG, 627 ± 73 pA, 15 cells/12 mice; Significant Interaction Genotype × Drug, F_(1, 10)_ = 13.64, *p* = 0.004, Uncorrected Fisher’s LSD, *p* = 0.02; Fig. 5C).

DB in CA1Ps is more likely to occur in cells proximal to the CA2 compared to those in the distal portion (Knauer and Yoshida, 2019). To ensure that our results were not influenced by sampling bias, we compared the number of cells exhibiting DB and persistent firing in the proximal and distal portions of CA1 and found no significant difference between genotypes, indicating that sampling bias was not a factor (χ^2^_=_ 1.5, *p* = 0.22). Exposure to 20 μM CCh significantly decreased DB current in WT cells as well but had no effect on DB current in GCG cells (Significant Drug Effect, F_(1, 9)_ = 6.903, *p* = 0.03, Uncorrected Fisher’s LSD, WT, *p* = 0.01, GCG, *p* = 0.6, WT vs GCG 20 μM CCh, *p* = 0.04; Supp. Fig. 4E). However, DB current during 20 μM CCh exposure in GCG cells remained significantly higher than CCh-induced DB current in WT cells, mirroring the effect observed during 5 μM CCh exposure (Significant Effect of Drug, F_(1, 9)_ = 3.302, *p* = 0.03, Uncorrected Fisher’s LSD, *p* = 0.04; Supp. Fig. 4E).

These results suggest that cholinergic modulation of DB is impaired in GCG neurons limiting regulation of excessive excitation. Thus, this disruption may contribute to altered excitability and increased seizure susceptibility in this model of epilepsy.

### 3.5. Muscarinic receptor activation modulation of CA1P excitability is preserved

Cholinergic signaling in the brain occurs through two main receptor families: muscarinic and nicotinic receptors. Muscarinic receptor activation is primarily responsible for ACh-induced depolarization of CA1Ps (Benardo and Prince, 1982b, 1982c; Scuvée-Moreau et al., 1997; Strømme et al., 2002) and is associated with seizures and epilepsy (Cruickshank et al., 1994; Hamilton et al., 1997; Turski et al., 1989). Thus, we first studied the effects of selective activation of mAChRs on CA1Ps in GCG mice.

**Fig. 6.**
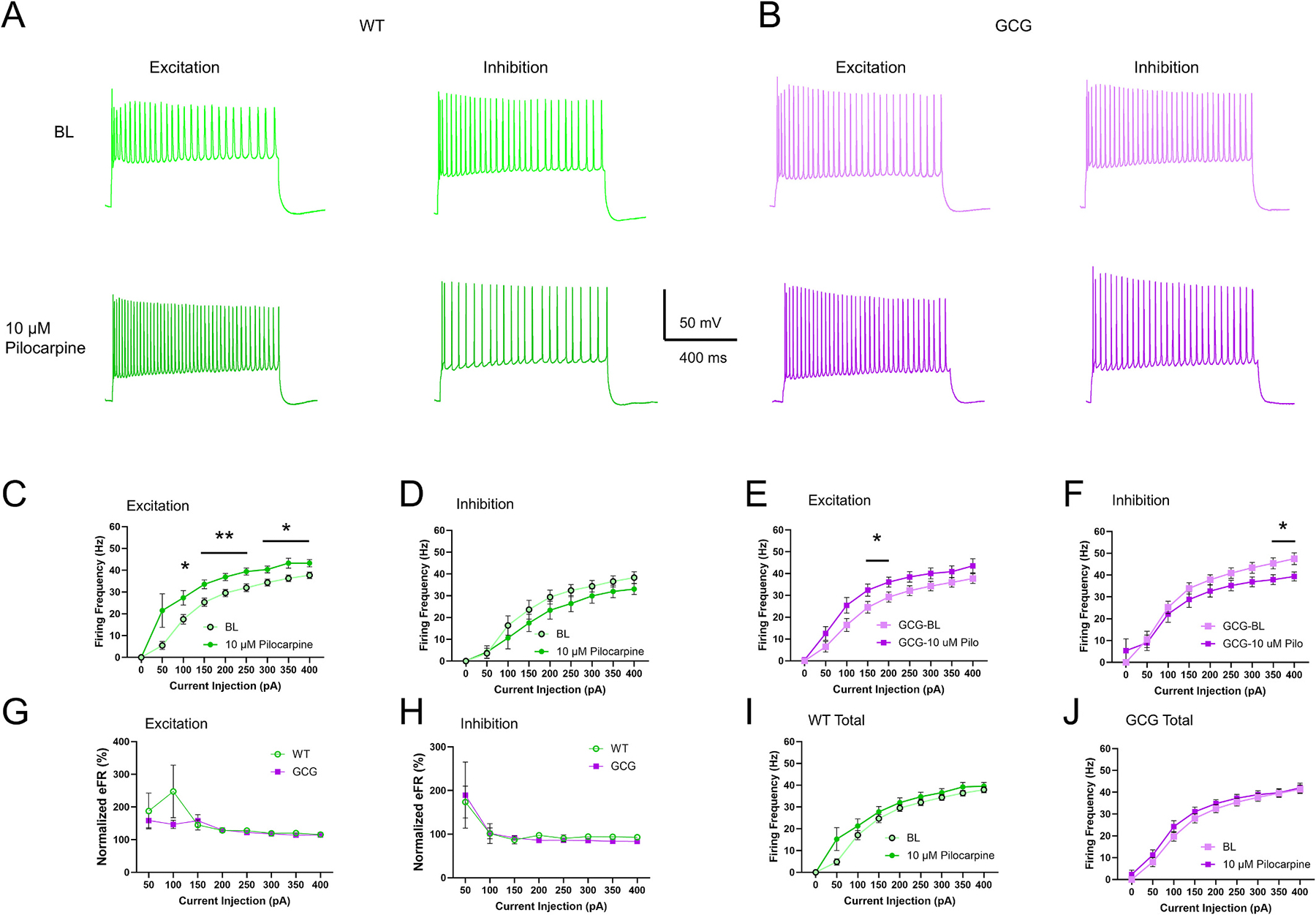
Pilocarpine bidirectionally modulates CA1 pyramidal cell excitability. (A, B) Representative traces of increased and decreased excitability in CA1Ps from WT and GCG mice, respectively, in response to 10 μM pilocarpine. (C, E) Firing frequency plots showing increased excitability in WT and GCG CA1Ps during depolarizing current injections (WT: n=16 cells/11 mice; GCG: n=20 cells/8 mice). (D, F) Firing frequency plots showing decreased excitability in WT and GCG CA1Ps during depolarizing current injections (WT: n=9 cells/8 mice; GCG: n=12 cells/6 mice). (G, H) Normalized firing frequency plots of WT and GCG CA1Ps that exhibited excitation and inhibition, respectively, in response to 10 μM pilocarpine. (I, J) Total firing response plots for WT and GCG CA1Ps (WT: n=25 cells/12 mice; GCG: n=32 cells/10 mice). Data are presented as mean ± SEM. Statistical significance was determined using a two-way repeated-measures ANOVA with Tukey’s post-hoc test or mixed effects model for firing frequency. *P < 0.05, **P < 0.01, ***P < 0.001.

The general muscarinic receptor agonist pilocarpine (10 μM) elicited two different effects on eFR in both WT and GCG cells (Fig. 6 A–B). Pilocarpine increased the firing rate in some cells and decreased it in others, showing no overall effect when the groups are combined (WT Drug Effect, F_(1, 48)_ = 2.127, *p* = 0.15; GCG Drug Effect, F_(1, 62)_ = 0.8519, *p* = 0.36; Fig. 6C–H). As such, DB current was unaffected in both genotypes in the presence of pilocarpine (Supp. Fig. 5A–C). Similarly, membrane properties of the cells that increased in firing activity remained unchanged, similar to CCh’s effects (Table 2). WT cells that decreased firing activity also displayed changes in membrane properties consistent with decreased excitability including increased rheobase, decreased input resistance, and hyperpolarization of resting membrane potential, with no effects on time constant, sag or inward rectification (Table 2). GCG cells that decreased firing activity, however, had no differences in membrane properties (Table 2). When looking at the combined data, WT cells showed an increased rheobase and hyperpolarization of resting membrane potential, while GCG cells displayed hyperpolarization of the resting membrane potential (Table 2). Overall, aside from a difference in sag, pilocarpine administration did not result in significant genotype-dependent effects.

We also examined action potential properties. Both WT and GCG cells that exhibited increased firing activity showed changes in action potential properties consistent with increased excitability and faster firing kinetics including decreases in action potential amplitude, half-width, overshoot, and decay time, along with significant hyperpolarizations of the thresholds. Both WT and GCG cells that exhibited decreased firing activity displayed fewer action potential changes. WT cells showed an increase in mAHP, while other properties remained unchanged. GCG cells showed no effects on action potential properties.

These data suggest that pilocarpine-induced hypoexcitability compared to hyperexcitability is associated with fewer compensatory modifications in action potential properties. When analyzing the combined data, both WT and GCG cells demonstrated similar significant decreases in action potential amplitude, half-width, overshoot, and threshold hyperpolarization. In contrast, changes in decay time and an increase in mAHP were observed in WT cells, but these properties remained unaffected in GCG cells (Table 2). Together, these findings indicate that though there were some subtle differential effects of pilocarpine based on genotype, the mAChR signaling is relatively preserved despite *Arx* dysfunction.

### 3.6. Nicotinic receptor-induced increase in excitability is abolished

Although postsynaptic cholinergic effects of ACh on CA1Ps have been demonstrated to be mediated primarily through mAChRs, nicotinic receptors (nAChRs) regulate CA1P excitability as well (Chung et al., 2016; Chung and Bailey, 2018). Thus, we assessed whether nicotinic signaling was altered in GCG cells. We found that 10 μM nicotine increased firing activity of CA1Ps in WT mice but had no effect on GCG CA1P firing activity indicating dysfunctional signaling (Significant Interaction Current Injection × Drug, F_(8, 272)_ = 2.422, *p* = 0.02; Fig. 6). DB current was unaffected in both genotypes (F_(1,12)_ = 0.5798, *p* = 0.46; Supp. Fig. 5D). Though resting membrane potential trended towards depolarization and sag trended towards a decrease, no significant effects on membrane properties were observed (Table 3). In WT cells, action potential properties changed in a manner consistent with faster firing kinetics including significantly decreased amplitude, along with half-width, overshoot, and decay time (Table 3). Additionally, action potential threshold was slightly hyperpolarized while rise time, fAHP, overshoot, and mAHP remained unaffected. In GCG cells, all action potential properties were unaffected except for fAHP, which decreased (Table 3). These results suggest *Arx* dysfunction disrupts nAChR signaling in CA1Ps.

### 3.7. AChR expression in the hippocampus is altered

Together, the reduction in ChAT expression, an altered threshold for CCh-induced DB, and a disrupted functional response to nAChR activation, led us to hypothesize that cholinergic receptor expression changes underly these electrophysiological alterations. Cholinergic signaling in the brain is mediated by two primary receptor families: mAChRs (M1, M2, M4) and nAChRs (α3β4, α4β2, and α7). Therefore, we compared the protein expression of AChR subunits in micro dissected regions of the hippocampus of WT versus GCG mice using western blot.

**Fig. 7.**
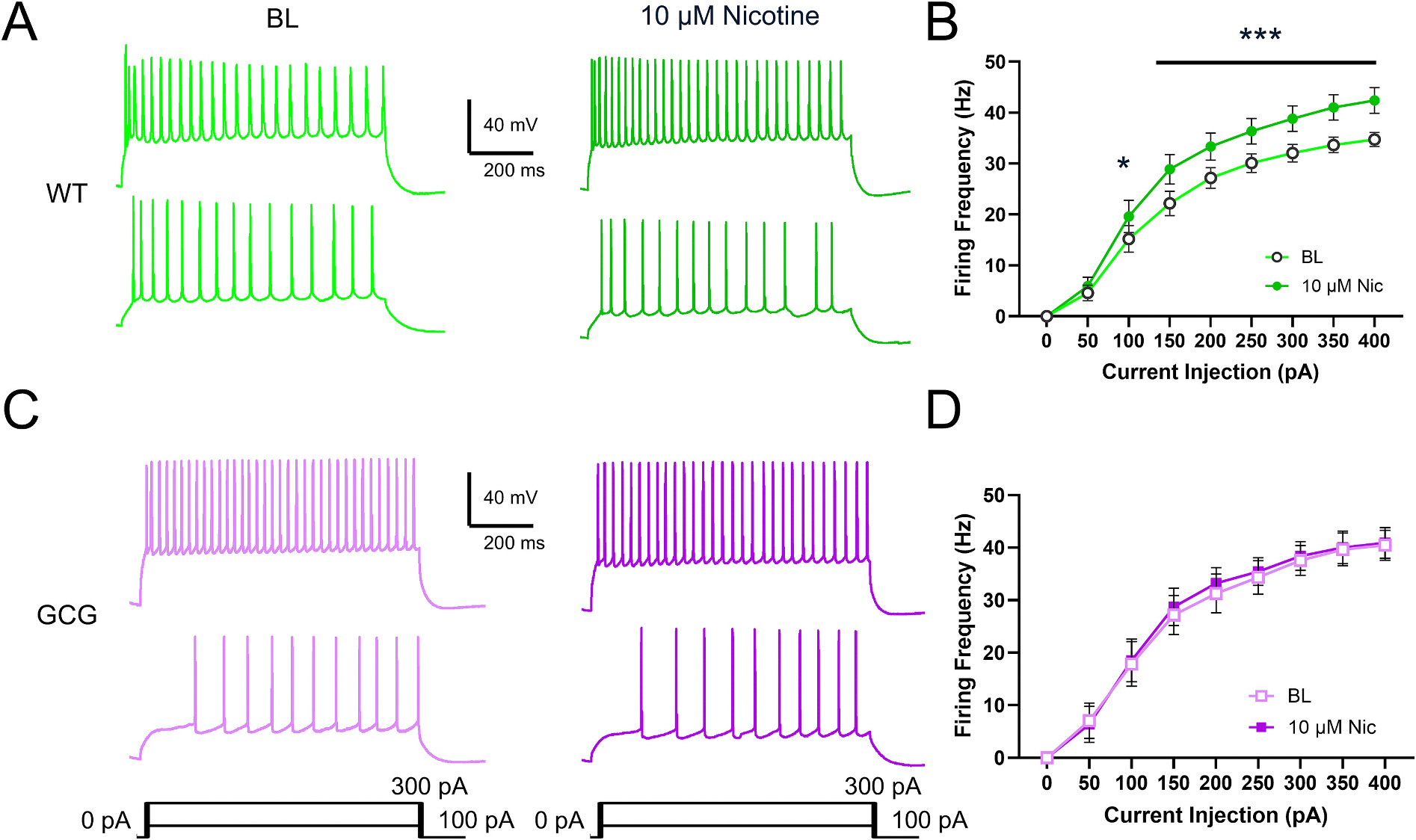
Nicotinic excitability enhancement is impaired in GCG mice. (A, C) Representative traces of evoked firing activity of CA1Ps before and during 10 μM Nicotine exposure from WT and GCG mice, respectively. (B, D) Plots of firing frequency in response to 50 pA depolarizing current steps before and during carbachol administration in WT and GCG mice, respectively. Data are presented as mean ± SEM (WT, n=18 cells/8 mice; GCG, n=12 cells/5 mice). Statistical significance was determined using a two-way repeated-measures ANOVA with Tukey’s post-hoc test or mixed effects model for firing frequency. *P < 0.05, **P < 0.01, ***P < 0.001.

**Fig. 8.**
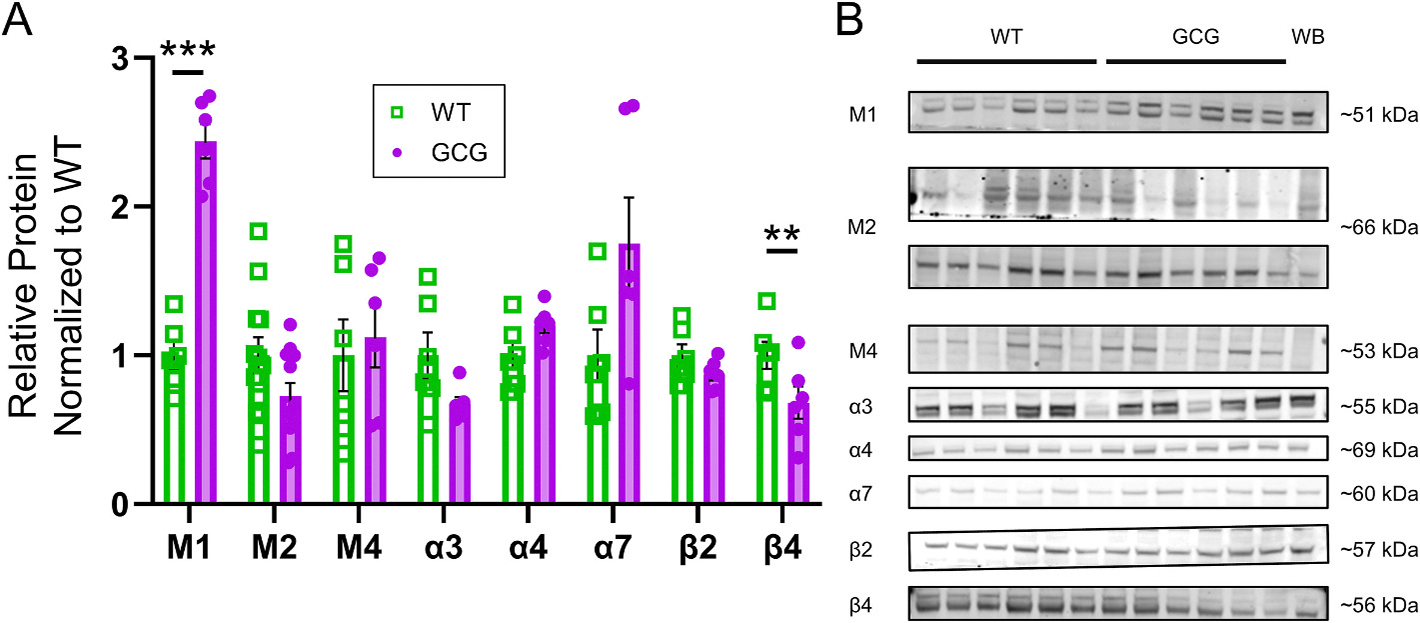
Cholinergic receptor expression is altered in the CA1 region of the hippocampus in GCG mice. (A) Quantification of western blot data showing cholinergic receptor expression. Target protein levels were normalized to total protein. (B) Representative western blot images of muscarinic receptors (M1, M2, M4) and nicotinic acetylcholine receptor subtypes (α3, α4, α7, β2, β4). Data are presented as mean ± SEM (WT, n=6 or 12; GCG, n=6). Statistical significance was determined using unpaired t-tests. *Q < 0.05, **Q < 0.01, ***Q < 0.001. WB= whole brain.

In CA1, M1 expression was increased (WT, 1, *n* = 6, GCG, 2.4, n = 6, *q* < 0.001; Fig. 8) and β4 expression was decreased (WT, 1, n = 6, GCG, 0.3 n = 6, *q* = 0.006; Fig. 8). α4 (WT, 1, n = 6, GCG, 1.2 n = 6, *q* = 0.08; Fig. 8) and α7 (WT, 1, n = 6, GCG, 1.8 n = 6, *q* = 0.08; Fig. 8) trended towards an increase in expression and α3 (WT, 1, n = 6, GCG, 0.6 n = 6, *q* = 0.08; Fig. 8) and M2 (WT, 1, *n* = 12, 6 per blot, 2 blots, GCG, 0.7 n = 12, 6 per blot, 2 blots, *q* = 0.08; Fig. 8) trended towards a decrease, but were not significantly different (Fig. 7). We also assessed AChR expression changes in the DG as a means to understand whether changes are unique to hippocampal subregions or are more widespread. In the DG, M1 (WT, 1, n = 12, 6 per blot, 2 blots, GCG, 0.7, n = 12, 6 per blot, 2 blots, *q* = 0.14; Supp. Fig. 6) and β4 (WT, 1, n = 6, GCG, 0.7, n = 6, *q* = 0.14; Supp. Fig. 6) trended towards a decrease, though did not reach significance while M2, M4, α3, α4, α7, and β2 expression levels remained the same (Supp. Fig. 6). Together, these findings suggest that *Arx* disruption changes AChR expression in the hippocampus, primarily in the CA1 subregion.

## 4. Discussion

In this study, we demonstrate that despite significantly diminished cholinergic presence in the basal forebrain, subtle physiological alterations occur in the GCG mice in response to cholinergic activation suggesting that the diminished cholinergic presence may alter hippocampal network dynamics, contribute to seizure susceptibility, and cognitive impairment leading to the development of epilepsy and intellectual disability (ID) in the *Arx*-related epilepsy and ID model. This is the first study to examine the role of cholinergic dysfunction in a model of a DEE. Immunohistochemical analysis confirmed markedly decreased ChAT expression in the basal forebrain of GCG mice, as reported by Kitamura et al. (2009). Patch-clamp electrophysiological recordings demonstrated modest hyperexcitability of CA1Ps accompanied by a decrease in sag current in GCG mice. Interestingly, CCh modulation of CA1P firing activity was preserved in GCG as in WT mice. However, CCh-induced DB current was significantly increased in GCG cells. Selective activation of mAChRs, which primarily underlies CCh mediated effects on CA1Ps, affected neuronal excitability similarly in both genotypes, though with two different responses. By contrast, selective activation of nAChRs, the other class of AChRs, failed to increase excitability of GCG CA1Ps as it did in WT ones. In an effort to uncover AChR reorganization and/or upregulation as a homeostatic response to decreased cholinergic input, we observed subtle changes in AChR subtype expression, particularly in M1 and β4. Together, we demonstrate that cholinergic changes induced by Arx dysfunction lead to non-cell-autonomous alterations in CA1 pyramidal neuron intrinsic excitability, accompanied by possible homeostatic adjustments in receptor expression (See summary figure).

### 4.1. ChAT expression is decreased in the basal forebrain

We confirmed the reduction in ChAT expression in the MS and DBB of GCG mice as reported by Kitamura et al. (2009). Our study explores the electrophysiological and receptor expression consequences of this change. We also found that NeuN staining a marker of neurons was not altered, suggesting that either the ChAT expressing cells were developmentally reprogramed to express another neurotransmitter or were not present but NeuN staining was not sensitive enough to pick this up. Studies on *Lhx8, Gbx1, Isl1* and *Arx*, key regulators of cholinergic neuron development have been performed. Arx regulates *Lhx7/8* and *Gbx1*, both of which are reduced in *Arx*-deficient mice (Colasante et al., 2009; Colombo et al., 2007). Consequently, cholinergic neurons of different populations are also reduced (Colombo et al., 2007). Notably, *Lhx8*^−/−^ mice exhibit reduced *Arx* expression in the subventricular zone (SVZ) of the medial ganglionic eminence (Flandin et al., 2011), and *Arx* deficiency leads to increased *Lhx8* in the SVZ but reduced expression in the mantle zone by E12.5 (Colombo et al., 2007). Additionally, *Isl1* regulates *Arx* in pancreatic alpha cells, suggesting a broader role in cell fate determination. This current knowledge of ChAT precursors development supports the idea that *Arx* mutations disrupt cholinergic cell fate determination rather than migration or induce cell death.

Supporting this idea is that NeuN expression in MS and DBB of GCG was not decreased and there is little evidence of widespread cell death in the GCG brain (Siehr et al., 2020). The proportion of cholinergic cells in the basal forebrain (9000 cholinergic neurons ~2 % of basal forebrain neurons (Peterson et al., 1999) is maybe too small to significantly impact the total neuron count. Together, we believe, the literature and our data support the notion that cholinergic neurons of the basal forebrain fail to develop properly in the Arx mouse model. Future studies could examine alternative cholinergic markers such as *Lhx8*, *Gbx1*, and *Isl1*, or p75, a neurotrophin receptor highly expressed in basal forebrain cholinergic neurons to determine what fates these cells take in the absence of Arx.

### 4.2. Cholinergic system dysfunction impacts network excitability

The CA1 is the major output of the hippocampus and thus is critical for understanding the ultimate effects on the hippocampal network. First, we determined the baseline excitability of CA1Ps in the GCG mice compared to WT mice and demonstrated that CA1P firing activity is modestly yet significantly elevated in GCG cells at the upper range of current injections. Overall, including lower current injections, there was no genotype difference. The increased firing rate in the more depolarized neurons likely reflects biologically meaningful shifts in excitability thresholds, that related to the generation of seizures and cognitive differences in these GCG mice.

Beguin et al. (2013) also investigated the firing activity of CA1Ps in GCG mice but at a younger age (p14) (Beguin et al., 2013) and did not find differences in firing rates. This discrepancy may be due to our using higher current injections, as we did not find changes in excitability in the current injection range of their study or the difference could be due to the ages studied (p14 vs p30) as the hippocampal network is continuing to develop across this age-span.

Hyperexcitability of CA1Ps at p30+ has been reported in another *Arx* mutant mouse model (Joseph et al., 2024). In a conditional knockout model where *Arx* is deleted from parvalbumin-expressing interneurons postnatally, CA1Ps exhibit hyperexcitability along with altered membrane properties, including a decreased sag ratio (Joseph et al., 2024). Similarly, in GCG mice, CA1P hyperexcitability is accompanied by a reduction in sag current, suggesting impaired HCN channel function or decreased HCN expression. In addition, these findings are consistent with previous reports linking HCN channel dysfunction to epilepsy (Arnold et al., 2019; Kessi et al., 2022). In particular, in a temporal lobe epilepsy rat model, HCN expression was reduced in the dorsal CA1, which was associated with hyperexcitability (Arnold et al., 2019).

In the conditional *Arx* parvalbumin-KO model, CA1P hyperexcitability was attributed to increased voltage-dependent sodium channel activity, a mechanism that may also contribute to excitability in GCG mice. Additionally, Mattiske et al. (2016) found significant upregulation of potassium (K^+^) channel genes in the telencephalon of 12.5 days post conception GCG mice, with *Kcna3*, *Kcnq3*, and *Kcnj3* increasing 2.5-, 2-, and 1.7-fold, respectively (Mattiske et al., 2016). These channels play key roles in repolarizing the membrane after an action potential and regulating neuronal excitability via mechanisms such as the M-current and inward rectification, which influence how neurons integrate synaptic inputs and maintain stability. While increased K^+^ channel expression might counteract hyperexcitability under normal conditions, it is unclear whether these changes are a direct consequence of *Arx* dysfunction, a compensatory response, or even persist into adulthood.

Overall, GCG CA1Ps are modestly hyperexcitable on a cellular level, particularly when more depolarized which may be amplified on the network level. We focused on intrinsic properties at P30+, a developmental stage when the hippocampal network is functionally mature and GCG mice begin to exhibit frequent seizures, future studies could investigate earlier time points to better understand the developmental processes leading to seizure onset or older ages to determine how hyperexcitable CA1Ps become.

### 4.3. Cholinergic modulation of CA1P excitability

We found that cholinergic modulation of CA1Ps was similar in GCG and WT mice. This was somewhat unexpected, as studies have shown that loss of cholinergic input to the hippocampus leads to altered cholinergic receptor expression (Levey et al., 1995; Roßner et al., 1995; Wall et al., 1994), changes in synaptic and network activity (Kanju et al., 2012; Lee et al., 1994; Miller et al., 1994; Mizumori et al., 1990; Sainsbury and Bland, 1981), and even deficits in hippocampal-dependent behaviors such as learning and memory (Fragkouli et al., 2005; Kanju et al., 2012; Martyn et al., 2012; Mizumori et al., 1990; Moreau et al., 2008; Motooka et al., 2001) including in Alzheimer’s studies (Chen et al., 2022; Hampel et al., 2019; Hampel et al., 2018; Stanciu et al., 2019). Importantly, however, none of these prior studies directly measured cellular responses to cholinergic agonists. Our findings, therefore, provide the first direct evidence that CA1Ps in mutant and WT mice respond similarly to cholinergic modulation.

Given the significant reduction in ChAT expression in hippocampal-projecting basal forebrain cholinergic neurons in GCG mice, one would expect significant alterations in hippocampal cellular electrophysiological properties. Kitamura et al. (2009) reported an ~80 % decrease in hippocampal AChE, which is expressed in cholinergic terminals indicating reduced cholinergic input into the hippocampus. Despite this reduction in endogenous input, our findings indicate that cholinergic modulation of CA1Ps persists, suggesting a compensatory mechanism to keep ACh receptors present and responsive.

In support of our findings, there is evidence that as much as 40 % of ACh content may remain in the hippocampus after near complete loss of basal forebrain cholinergic neurons, indicating a partial compensatory mechanism (Chang and Gold, 2004; Dornan et al., 1997; Lapchak et al., 1991; Mcmahan et al., 1997). There’s little to no evidence of functional cholinergic interneurons in the hippocampus (Blusztajn and Rinnofner, 2016; Yi et al., 2015) and thus are unlikely to compensate for decreased MS and DBB input. Finally, although we did not measure ACh levels in the hippocampus of GCG mice and it was not reported by Kitamura et al. (2009), it remains possible that GCG mice retain a functional level of ACh in the hippocampus therefore are able to modulate firing rate of CA1Ps the same as in WT. It is possible that ACh release is upregulated in existing terminals in GCG mice.

Our study also provides a direct contrast to well-characterized models of cholinergic deafferentation, such as those observed in Alzheimer’s disease. In Alzheimer’s disease models, degeneration of basal forebrain cholinergic neurons leads to reduced intrinsic excitability and diminished ACh responsiveness in downstream targets like prefrontal layer 6 neurons (Proulx et al., 2015) as well as a transient upregulation of nicotinic responses at mid-disease stages, (Power et al., 2024). Similarly, one might expect CA1Ps to exhibit reduced excitability and increased nicotinic sensitivity following cholinergic loss. However, our findings diverge sharply from these expectations.

### 4.4. Depolarization block

While CCh did not differentially modulate firing rates in GCG neurons, we did observe a difference in the threshold of DB. In addition, our findings reveal a dose-dependent modulation of DB by CCh in GCG neurons that differs from WT. Specifically, 5 μM CCh increased the DB threshold in GCG cells, whereas 20 μM CCh had no significant effect. The absence of a further increase at the higher dose may reflect a ceiling effect, where the mechanisms regulating DB in GCG neurons are saturated or impaired, limiting additional modulation by cholinergic stimulation. DB is a neuronal property that occurs throughout the central nervous system, is associated with epilepsy and may act as a protective mechanism against neuronal overexcitation (Goldensohn and Purpura, 1963; Phelan et al., 2012). Dysfunction of DB may contribute to seizure dynamics by either prolonging excessive firing or impairing inhibitory mechanisms. The underlying mechanisms under baseline conditions include lack of deinactivation of transient sodium channels (NaT), decreased NaT density and incomplete deinactivation of delayed rectifier potassium channels (Kdr) (Bianchi et al., 2012; Colombo et al., 2007; Tucker et al., 2012).

CCh is also known to induce DB through unique pathways in CA1Ps by acting on TRPC, M, and SK channels in CA1Ps (Fraser and MacVicar, 1996; Knauer and Yoshida, 2019; Tai et al., 2011). Under cholinergic stimulation, mAChR receptor agonism activates TRPC channels, likely via PLC, to produce a depolarizing non-selective cation conductance while simultaneously suppressing K^+^ currents, notably M and SK channels. This combination of enhanced inward cationic current and reduced outward K^+^ conductance increases net depolarizing drive, which inactivates voltage-gated Na^+^ channels, leading to DB. In fact, TRPC activation alone is sufficient to induce DB (Knauer and Yoshida, 2019). This also aligns with growing literature linking TRPC channels to epilepsy (Yu et al., 2022). The impaired CCh-induced DB suggests that one or more of the mechanisms, potentially TRPC, or downstream signaling cascades are dysfunctional.

Given that GCG mice have spontaneous seizures, impaired DB may be a contributing factor to network excitability and seizure generation. Furthermore, in this context, DB may fail to serve as an impediment to overexcitation, thus, leading to seizure activity. We hypothesize that TRPC-mediated mechanisms are impaired in GCG neurons, as the DB threshold was unchanged under baseline conditions, when DB is primarily mediated by NaT and KDr channels, but shifted only during carbachol application, a condition known to engage TRPC-dependent pathways. Therefore, DB mechanisms may serve as potential targets for therapeutic intervention. Future studies should investigate the mechanisms underlying impaired DB in GCG mice.

### 4.5. Muscarinic receptor modulation of CA1Ps

Muscarinic receptor activation primarily mediates ACh’s effects on CA1Ps (Benardo and Prince, 1982b, 1982c; Scuvée-Moreau et al., 1997; Strømme et al., 2002). In our study, we observed a modulatory effect of the selective mAChR agonist pilocarpine, although there was no differential effect between genotypes, similar to CCh. However, pilocarpine elicited two opposing effects, excitatory and inhibitory. Several studies have demonstrated that cholinergic receptor activation produces differential effects on CA1Ps, which can be influenced by anatomical location or firing type such as early bursting vs. late bursting (Graves et al., 2012; Tsotsokou et al., 2024). Although chronic excitation and chronic inhibition are not commonly reported, some studies demonstrate that transient AChR activation can lead to initial hyperpolarization followed by depolarization in approximately 50 % of CA1Ps (Benardo and Prince, 1982a; Dasari and Gulledge, 2011; Gulledge and Kawaguchi, 2007; Wakamori et al., 1993). Though we used tonic administration of CCh in our study and observed persistent rather than phasic hyperpolarization, roughly one third of the cells were inhibited by pilocarpine.

It is unclear why we observed persistent rather than phasic hyperpolarization in our study, but several possible explanations exist. Perhaps because the bath administration of pilocarpine in our study is a slower mechanism than transient focal application as performed in many of the aforementioned studies, this may have also translated to a slower and longer lasting inhibitory effect as well. It is also possible that a subset of CA1Ps in GCG mice may have an increased expression of inhibitory Gi-coupled mAChRs (M2 and M4) rather than excitatory Gq-coupled receptors (M1 and M3), which are the primary mediators of mAChR activation in CA1Ps (Dasari and Gulledge, 2011). Alternatively, a subpopulation of CA1Ps may receive stronger inhibitory input that is modulated by mAChR activation (Goswamee and McQuiston, 2019).

Another explanation is that the biphasic effects observed could be specific to pilocarpine itself. Interestingly, a previous study reported no effect of pilocarpine on field activity in the CA1, which is similar to our findings when the two groups of effects were not analyzed separately (Marchi et al., 2007). In pilocarpine-induced epilepsy rodent models, CA1P membrane properties are relatively preserved (Postnikova et al., 2024). Pharmacological experiments using subtype-specific antagonists could help delineate the receptor contributions to these responses. Additionally, blocking inhibitory synaptic transmission may clarify the role of GABAergic input, while paired recordings or optogenetic approaches could provide insights into how local circuitry and connectivity influence the observed excitatory and inhibitory responses. These findings highlight the complexity of mAChR activation in the hippocampus.

### 4.6. Nicotinic receptor modulation of CA1Ps

Although postsynaptic nAChR modulation of CA1Ps had been previously debated, studies have demonstrated that these receptors are indeed present and that their activation enhances excitability (Chung et al., 2016; Chung and Bailey, 2019; Chung and Bailey, 2018; Ji et al., 2001; Kalappa et al., 2010). This modulatory effect is developmentally regulated, being most pronounced between postnatal days 7 and 14 and significantly reduced after p30, although still detectable (Chung et al., 2016). Our findings are consistent with these observations in WT cells. We found that activation of nAChRs using 10 μM nicotine on CA1Ps increased neuronal excitability in WT cells, whereas no effect was observed in GCG cells. One possible explanation is that, in GCG mice, the translation or transcription of nAChRs is reduced; however, our protein data only partially support this hypothesis, namely, through a decrease in β4 subunit expression. Alternatively, alterations in post-translational modifications may contribute to the observed differences.

The diminished response to nAChR activation at later developmental stages, after P30, suggests that receptor function may also be dysregulated developmentally, which could have downstream consequences including impaired synaptic physiology. Because general AChR and selective mAChR agonism fail to elicit differential effects on CA1Ps from GCG mice when nAChR agonism does, this supports previous evidence that the mechanism of AChR agonism is via mAChRs. Perhaps, dysfunctional nAChR modulation is more consequential to developmental processes. Future studies will aim to elucidate the mechanisms underlying this developmental regulation and its impact on neural circuitry.

### 4.7. Cholinergic receptor expression

The cholinergic system and specifically, cholinergic receptor dysfunction is strongly implicated in epileptogenesis (Wang et al., 2021; Wang et al., 2020). Sleep-related hypermotor epilepsy is caused by gain-of-function mutations in nAChR subtypes *CHRNA4*, *CHRNB2*, and *CHRNA2* and mutations in *PRIMA1*, which encodes a protein that secures acetylcholinesterase to the membrane (Aridon et al., 2006; Hildebrand et al., 2015; Phillips et al., 2001; Steinlein et al., 1997). Decreased α7 nAChR can increase seizure susceptibility (Sun et al., 2021). Pilocarpine-treated rodent models have been widely used in epilepsy research (Turski et al., 1989) and the M1 receptor has been reported to have pro-convulsive effects (Hamilton et al., 1997).

We examined cholinergic receptor expression in the hippocampus, expecting to observe changes similar to those reported in lesion studies. Our results showed a decrease in the β4 subunit in CA1 and an increase in M1 in the CA1 along with subtle trends in other subtypes in the CA1 and DG. The utilization of microdissected hippocampal tissue, which include pyramidal cells and interneurons, inhibits our ability to tease out changes in a cell type specific manner. We also cannot exclude the possibility that receptors change in opposing directions in different cell types therefore masking cell specific effects.

Lesions of basal forebrain cholinergic neurons have been reported to cause significant upregulation of M1, M3, and M5 receptors in the hippocampus, while M2 expression decreases or remains unchanged (Levey et al., 1995; Wall et al., 1994). These changes are time-dependent, as receptor expression levels fluctuate between 3 days and 28 days post-lesion (Levey et al., 1995). Similarly, Alzheimer’s patients have altered levels of M1 receptors (Flynn et al., 1995). The M1 receptor in particular has been highly implicated in epileptogenesis and may also contribute in *GCG* as well. As evidence indicates that M1 receptor agonism is pro-convulsive (Hamilton et al., 1997), our study demonstrates that M1 receptor expression is upregulated in the CA1 of *GCG* mice as a compensatory mechanism, but this may increase their susceptibility to seizures and impair their neurodevelopment.

Notably, β4 was the only nAChR subtype to show significant changes in expression in response to basal forebrain cholinergic neuron loss, consistent with lesion studies (Roßner et al., 1995). However, chronic nicotine exposure has been shown to upregulate and desensitize nAChRs (Dani et al., 2000; Marks et al., 1992; Nashmi and Lester, 2007; Pidoplichko et al., 1997). In our study, we observed a downregulation of the β4 subunit, which is co-expressed with α3. The α3β4 nAChRs are primarily located in glutamatergic inputs to CA1 (Alkondon and Albuquerque, 2002). Though we did not record synaptic input, Beguin et al. (2013) reported increased excitatory input to CA1Ps. Thus, the increased α3β4 nAChR expression may account for the increased glutamatergic drive reported by Beguin et al. (2013). Together, changes in AChR expression in the CA1 may underly excitability alterations and consequently, seizure susceptibility in GCG mice.

## 5. Conclusion

Our findings reveal a homeostatic cholinergic compensation model in which reduced ChAT expression leads to diminished cholinergic tone in the hippocampus. The extent of developmental compensation, in the setting of >60 % loss of ChAT input to the hippocampus, was unexpected. In response to this deficit, CA1Ps exhibit modestly elevated firing activity under strong input, reflecting a shift towards hyperexcitability. At the receptor level, two compensatory changes emerge: upregulation of M1 mAChRs and downregulation of β4-containing nAChRs. While increased M1 expression preserves responsiveness to muscarinic agonists such as pilocarpine, this adaptation fails to fully restore normal cholinergic function. One possibility is that receptor upregulation is offset by impaired downstream signaling or incomplete coupling to ion channel targets, limiting the ability of M1 activation to regulate excitability. Notably, the threshold for carbachol-induced DB is significantly elevated, suggesting dysfunction in downstream mechanisms such as the PLC pathway. This also indicates that an important inhibitory mechanism, normally engaged to suppress pyramidal neuron firing during excessive depolarization, is weakened and may contribute to altered synaptic integration and excitatory drive, as previously demonstrated by Beguin et al. (2013).

Together, the cumulative effect of the subtle changes we uncovered suggest that the hippocampal network dynamics are altered and contribute to seizure susceptibility, cognitive impairment, and developmental abnormalities in GCG mice (see summary figure). These finding, diverge from the expectations of large effects on the hippocampal network with the dramatic change in cholinergic projections to the hippocampus. Future, work will study the network level changes in these GCG mice to support targeting the cholinergic system as a therapeutic strategy for Arx-related disorders and other DEEs.

## Supplementary Material

Supplementary Material 1

Supplementary Material 2

## Figures and Tables

**Table 1:** Comparison of effects of 5 μM Carbachol on CA1P Intrinsic Properties.

	WT Baseline (Mean ± SD)	WT Drug (Mean ± SD)	*p*-value (WT)	GCG Baseline (Mean ± SD)	GCG Drug (Mean ± SD)	p-value (GCG)	p-value (WT vs GCG, Baseline)	p-value (WT vs GCG, CCh)

Resting Membrane Potential (mV)	−64.63 ± 4.11	−61.84 ± 10.50	0.12	−66.68 ± 5.07	−65.44 ± 8.90	0.47	0.37	0.13
Input Resistance (MΩ)	171.00 ± 41.86	173.59 ± 62.73	0.86	169.94 ± 39.70	195.84 ± 76.25	0.09	0.95	0.21
Inward Rectification Ratio	1.12 ± 0.10	1.17 ± 0.16	0.32	1.09 ± 0.12	1.09 ± 0.16	1.00	0.49	0.09
Sag (mV)	6.87 ± 3.73	5.80 ± 3.03	*0.02	6.11 ± 2.22	5.05 ± 2.93	*0.02	0.42	0.42
Rheobase (pA)	75.45 ± 35.95	92.38 ± 45.82	0.91	77.27 ± 99.15	80.00 ± 66.63	0.44	0.41	0.89
Amplitude (mV)	82.69 ± 14.72	75.17 ± 15.17	*0.01	84.51 ± 15.92	72.79 ± 15.20	*** **< 0.0001**	0.60	0.57
Threshold (mV)	−43.41 ± 8.15	−42.39 ± 10.18	0.73	−42.79 ± 5.86	−44.81 ± 7.93	0.28	0.72	0.55
Rise Slope (mV/ms)	187.94 ± 73.75	167.14 ± 70.25	0.07	175.74 ± 58.59	137.54 ± 49.94	**0.00**	0.53	0.12
Rise Time (ms)	0.42 ± 0.10	0.43 ± 0.11	0.56	0.47 ± 0.12	0.51 ± 0.10	0.08	0.08	*0.02
Half-Width (ms)	1.31 ± 0.28	1.27 ± 0.29	0.41	1.57 ± 0.42	1.53 ± 0.29	0.42	** **< 0.01**	** **< 0.01**
Overshoot (mV)	39.42 ± 14.64	32.83 ± 18.21	0.06	41.88 ± 15.31	26.62 ± 17.20	*** **< 0.0001**	0.50	0.33
Decay Slope (mV/ms)	−60.01 ± 18.09	−53.62 ± 19.90	*0.03	−52.51 ± 16.11	−46.83 ± 14.92	0.06	0.16	0.20
Decay Time (ms)	1.29 ± 0.34	1.36 ± 0.49	0.31	1.55 ± 0.48	1.51 ± 0.37	0.52	*0.048	0.25
fAHP (mV)	−4.20 ± 2.74	−3.98 ± 3.03	0.68	−5.86 ± 3.50	−6.59 ± 3.21	0.91	0.09	0.08
mAHP (mV)	−5.38 ± 2.03	−6.72 ± 2.74	*** **< 0.0001**	−5.19 ± 2.50	−7.38 ± 3.20	** **< 0.01**	0.27	0.54
n (Cells/mice)	22/18	22/18		21/15	21/15			

**Table 2:** Effects of 10 μM Pilocarpine on CA1 Intrinsic Properties.

Population	Property	WT Baseline (Mean ± SD)	WT Pilocarpine (Mean ± SD)	p-value (WT)	GCG Baseline (Mean ± SD)	GCG Pilocarpine (Mean ± SD)	p-value (GCG)	p-value (WT vs GCG, Baseline)	p-value (WT vs GCG, Pilocarpine)

Total	Resting Membrane Potential (mV)	−65.3 ± 3.9	−68.5 ± 6.4	*0.014	−66.3 ± 4.0	−68.6 ± 7.1	*0.04	0.52	0.94
	Input Resistance (MΩ)	180 ± 51	165 ± 65	0.22	178.8 ± 56.9	175.7 ± 83.0	0.78	0.94	0.53
	Inward Rectification Ratio	1.1 ± 0.1	1.1 ± 0.2	0.19	1.0 ± 0.1	1.1 ± 0.3	0.08	0.22	0.30
	Sag (mV)	7.2 ± 1.9	6.4 ± 2.2	0.07	6.6 ± 2.1	6.1 ± 2.6	0.21	0.27	0.57
	Rheobase (pA)	64 ± 40	102 ± 25	**0.007	71 ± 32	79 ± 58	0.54	0.67	0.17
	Amplitude (mV)	92.5 ± 11.4	85.4 ± 13.6	**0.002	86.6 ± 12.1	82.2 ± 12.8	*0.03	0.08	0.34
	Threshold (mV)	−47.3 ± 3.7	−48.9 ± 5.3	*0.02	−46.4 ± 4.3	−48.1 ± 5.5	**0.003	0.44	0.57
	Rise Slope (mV/ms)	202.4 ± 58.0	206.3 ± 72.9	0.73	177.2 ± 51.0	177.1 ± 62.9	0.99	0.13	0.08
	Rise Time (ms)	0.4 ± 0.1	0.4 ± 0.1	0.22	0.5 ± 0.1	0.4 ± 0.1	0.66	0.17	*0.04
	Half-Width (ms)	1.5 ± 0.3	1.3 ± 0.2	***0.0004	1.6 ± 0.3	1.5 ± 0.2	*0.02	0.34	*0.012
	Overshoot (mV)	45.2 ± 11.5	36.5 ± 14.9	***0.0004	40.2 ± 13.9	34.1 ± 12.5	**0.004	0.16	0.49
	Decay Slope (mV/ms)	−51.7 ± 11.9	−56.7 ± 14.5	0.12	−46.4 ± 10.1	−48.5 ± 13.6	0.45	0.11	0.02
	Decay Time (ms)	1.6 ± 0.3	1.3 ± 0.2	**0.004	1.7 ± 0.3	1.5 ± 0.3	0.12	0.31	**0.008
	fAHP (mV)	−5.1 ± 2.1	−4.6 ± 1.9	0.13	−5.0 ± 2.6	−4.6 ± 2.1	0.25	0.77	0.99
	mAHP (mV)	−6.0 ± 2.4	−7.3 ± 2.4	***0.001	−6.2 ± 2.6	−6.8 ± 2.5	0.08	0.84	0.45
	n (Cells/mice)	25/12	25/12		32/10	32/10			
Excitation	Resting Membrane Potential (mV)	−64.9 ± 4.3	−66.2 ± 5.6	0.36	−66.1 ± 4.3	−69.0 ± 7.1	*0.04	0.51	0.15
	Input Resistance (MΩ)	183 ± 53	188 ± 64	0.76	171.4 ± 61.6	184.0 ± 97.4	0.36	0.62	0.86
	Inward Rectification Ratio	1.1 ± 0.1	1.2 ± 0.2	0.09	1.0 ± 0.1	1.2 ± 0.3	0.07	0.70	0.64
	Sag (mV)	7.6 ± 1.9	7.0 ± 2.1	0.25	6.9 ± 2.3	6.1 ± 2.2	0.09	0.30	0.19
	Rheobase (pA)	51 ± 28	55 ± 46	0.69	74 ± 36	69 ± 48	0.55	0.10	0.31
	Amplitude (mV)	91.4 ± 11.2	82.1 ± 13.3	**0.003	89.9 ± 10.6	81.2 ± 14.0	**0.002	0.73	0.84
	Threshold (mV)	−47.3 ± 3.6	−50.2 ± 4.2	***0.001	−46.3 ± 4.4	−49.2 ± 6.0	***0.0002	0.56	0.53
	Rise Slope (mV/ms)	193.6 ± 54.8	193.5 ± 74.8	1.00	179.3 ± 48.2	177.7 ± 60.1	0.91	0.48	0.43
	Rise Time (ms)	0.4 ± 0.1	0.4 ± 0.1	0.30	0.5 ± 0.1	0.4 ± 0.1	0.92	0.96	0.42
	Half-Width (ms)	1.6 ± 0.3	1.3 ± 0.2	**0.001	1.7 ± 0.3	1.4 ± 0.2	***0.0009	0.44	0.29
	Overshoot (mV)	44.1 ± 11.9	30.6 ± 14.7	***0.0001	43.2 ± 12.9	32.1 ± 12.9	**** **< 0.0001**	0.91	0.97
	Decay Slope (mV/ms)	−48.9 ± 8.7	−55.3 ± 16.5	0.15	−46.0 ± 9.8	−50.0 ± 14.8	0.31	0.50	0.22
	Decay Time (ms)	1.6 ± 0.3	1.3 ± 0.3	**0.008	1.7 ± 0.3	1.5 ± 0.4	*0.02	0.33	0.14
	fAHP (mV)	−5.1 ± 2.3	−5.3 ± 1.8	0.56	−5.1 ± 2.7	−4.9 ± 2.3	0.77	0.76	0.92
	mAHP (mV)	−6.3 ± 2.4	−7.4 ± 2.2	*0.03	−6.2 ± 2.7	−6.7 ± 2.7	0.26	0.87	0.40
	n (Cells/mice)	16/11	16/11		20/8	20/8			
Inhibition	Resting Membrane Potential (mV)	−66.2 ± 2.9	−72.5 ± 7.8	**0.007	−66.6 ± 3.6	−68.0 ± 7.5	0.45	0.86	0.07
	Input Resistance (MQ)	174 ± 49	123 ± 44	**0.008	191.0 ± 48.3	161.9 ± 51.8	0.07	0.43	0.07
	Inward Rectification Ratio	1.1 ± 0.2	1.1 ± 0.2	0.53	1.0 ± 0.1	1.0 ± 0.2	0.79	0.09	0.25
	Sag (mV)	6.6 ± 1.8	5.4 ± 2.2	0.16	6.1 ± 1.5	6.1 ± 3.4	1.00	0.64	0.49
	Rheobase (pA)	87 ± 49	187 ± 130	**0.003	65 ± 26	93 ± 73	0.28	0.52	**0.008
	Amplitude (mV)	94.5 ± 12.1	91.3 ± 12.7	0.19	80.9 ± 12.8	83.9 ± 11.0	0.17	*0.02	0.17
	Threshold (mV)	−47.5 ± 4.1	−46.6 ± 6.5	0.28	−46.4 ± 4.5	−46.4 ± 4.1	0.99	0.61	0.94
	Rise Slope (mV/ms)	218.0 ± 63.3	228.9 ± 67.5	0.46	173.7 ± 57.4	176.1 ± 70.0	0.84	0.13	0.07
	Rise Time (ms)	0.4 ± 0.1	0.4 ± 0.1	0.38	0.4 ± 0.1	0.5 ± 0.1	0.34	0.28	*0.03
	Half-Width (ms)	1.4 ± 0.2	1.2 ± 0.1	0.07	1.5 ± 0.2	1.5 ± 0.2	0.43	0.48	**0.003
	Overshoot (mV)	48.5 ± 9.0	45.5 ± 11.6	0.35	32.4 ± 13.2	37.5 ± 11.4	0.18	*0.03	0.18
	Decay Slope (mV/ms)	−56.6 ± 15.5	−59.2 ± 10.3	0.53	−47.0 ± 11.0	−45.9 ± 11.5	0.78	0.08	*0.02
	Decay Time (ms)	1.5 ± 0.3	1.3 ± 0.1	0.17	1.5 ± 0.2	1.6 ± 0.2	0.31	0.67	**0.005
	fAHP (mV)	−5.0 ± 1.8	−3.8 ± 1.8	0.10	−4.9 ± 2.5	−4.1 ± 1.9	0.15	0.95	0.86
	mAHP (mV)	−5.6 ± 2.5	−7.1 ± 2.8	**0.003	−6.2 ± 2.4	−7.0 ± 2.2	0.10	0.63	0.93
	n (Cells/mice)	9/8	9/8		12/6	12/6			

**Table 3:** Effects of 10 μM Nicotine on CA1P Intrinsic Properties.

	WT Baseline (Mean ± SD)	WT Drug (Mean ± SD)	p-value (WT)	GCG Baseline (Mean ± SD)	GCG Drug (Mean ± SD)	p-value (GCG)	p-value (WT vs GCG, Baseline)	p-value (WT vs GCG, Nicotine)

Resting Membrane Potential (mV)	−65.3 ± 3.7	−67.1 ± 5.9	0.15	−71.5 ± 9.4	−71.0 ± 13.1	0.74	*0.047	0.20
Input Resistance (MΩ)	176.0 ± 47.4	174.4 ± 56.6	0.93	214.0 ± 56.8	217.1 ± 122.4	0.88	0.16	0.12
Inward Rectification Ratio	1.0 ± 0.1	1.0 ± 0.1	0.46	1.0 ± 0.1	1.1 ± 0.3	0.49	0.80	0.12
Sag (mV)	5.9 ± 2.8	5.1 ± 2.4	0.13	5.8 ± 2.7	4.5 ± 1.9	0.06	0.90	0.52
Rheobase (pA)	68 ± 30	76 ± 48	0.53	65 ± 26	85 ± 62	0.16	0.84	0.56
Amplitude (mV)	91.2 ± 12.8	78.7 ± 15.0	*** **< 0.0001**	82.3 ± 15.3	83.4 ± 16.7	0.86	0.20	0.41
Threshold (mV)	−45.5 ± 6.8	−47.9 ± 6.2	*0.02	−49.0 ± 7.4	−50.6 ± 9.5	0.35	0.18	0.35
Rise Slope (mV/ms)	151.2 ± 40.6	145.4 ± 57.1	0.42	154.0 ± 55.9	166.0 ± 67.2	0.34	0.89	0.39
Rise Time (ms)	0.6 ± 0.1	0.5 ± 0.1	0.11	0.5 ± 0.1	0.5 ± 0.1	0.11	0.46	0.37
Half-Width (ms)	1.9 ± 0.4	1.6 ± 0.5	*** **< 0.0001**	1.7 ± 0.4	1.6 ± 0.3	0.15	0.21	0.98
Overshoot (mV)	45.7 ± 11.0	30.8 ± 15.1	*** **< 0.0001**	33.3 ± 17.6	32.8 ± 20.8	0.60	0.20	0.24
Decay Slope (mV/ms)	−42.9 ± 13.7	−48.7 ± 23.1	0.09	−42.9 ± 12.0	−45.1 ± 14.7	0.66	0.99	0.55
Decay Time (ms)	1.9 ± 0.5	1.6 ± 0.6	**0.002	1.7 ± 0.4	1.7 ± 0.4	0.40	0.39	0.79
fAHP (mV)	−5.3 ± 3.6	−5.4 ± 3.5	0.80	−4.9 ± 3.1	−4.2 ± 2.4	**0.003	0.56	0.24
mAHP (mV)	−5.0 ± 2.7	−5.9 ± 2.5	0.08	−5.0 ± 3.8	−4.6 ± 2.7	0.08	0.28	0.27
n (Cells/mice)	18/8	18/8		12/5	12/5			

## Data Availability

Data will be made available on request.

## Appendix A. Supplementary data

Supplementary data to this article can be found online at https://doi.org/10.1016/j.expneurol.2025.115591.

## Glossary

ACh: acetylcholine
ACSF: artificial cerebrospinal fluid
AP: action potential
ARX: Aristaless Related Homeobox gene
BL: baseline
CA1Ps: CA1 pyramidal cells
CCh: Carbachol
ChAT: choline acetyltransferase
DB: depolarization block
DBB: diagonal band of Broca
DEE: Developmental and Epileptic Encephalopathies
DG: dentate gyrus
fAHP: fast afterhyperpolarization
ISS: Infantile Spasms Syndrome
Kdr: delayed rectifier potassium channels
mAChR: muscarinic acetylcholine receptor
mAHP: medium afterhyperpolarization
MS: medial septum
NaT: transient sodium channels
NDS: normal donkey serum
NGS: normal goat serum
nAChRs: nicotinic acetylcholine receptors
OCT: optimum cutting temperature
PBS: phosphate-buffered saline
TBST: Tri-Buffered Saline with 0.1 % Tween
WT: wild-type
