# Supplementary material for: Cholinergic modulation of hippocampal CA1 pyramidal cell excitability in ArxGCG+7 mice
Source: Exp Neurol. Author manuscript; Available in PMC 2026 May 7. (PMC13150844; doi:10.1016/j.expneurol.2025.115591)
Supplement: Supplementary Material 1 [file NIHMS2157859-supplement-Supplementary_Material_1.docx]

**Supplementary Figures**

**Supplementary Figure 1: NeuN expression is unchanged in the basal forebrain of GCG mice.**

**Supplementary Figure 1: NeuN expression is unchanged in the basal forebrain of GCG mice.**

(A, B) Immunofluorescence images of NeuN expression in the medial septum (MS) and Diagonal Band of Broca (DBB) of wild-type (WT) and *Arx*^GCG+7^ (GCG). (C) Quantification of NeuN+ cells in the MS and DBB, normalized to area. Data are presented as mean ± SEM (WT, n=6; GCG, n=6). Statistical significance was determined using an unpaired t-test. *P < 0.05, **P < 0.01, ***P < 0.001.

**Supplementary Figure 2: Carbachol decreases spike train AHP.**

**Supplementary Figure 2: Carbachol decreases spike train AHP.**

(A,B) Representative traces of afterhyperpolarizations (AHP) after spike trains before and during 5 µM carbachol exposure in WT and GCG cells, respectively. (C) Histogram of the effect of 5 µM carbachol on AHP in WT and GCG cells. (D) Histogram of the effect of 5 µM carbachol normalized to baseline. Data are presented as mean ± SEM (WT, n=18 cells/15 mice; GCG, n=15 cells/10 mice). Statistical significance was determined using a two-way repeated-measures ANOVA with Fisher’s LSD post hoc analysis. *P < 0.05, **P < 0.01, ***P < 0.001. BL, baseline, CCh, carbachol.

**Supplementary Figure 3: Carbachol decreases spike frequency adaptation (SFA) in CA1 pyramidal cells (CA1Ps) similarly in WT and GCG mice.**

**Supplementary Figure 3:** **Carbachol decreases spike frequency adaptation (SFA) in CA1 pyramidal cells (CA1Ps) similarly in WT and GCG mice.**
(A, B) Representative firing responses of CA1Ps from wild-type (WT) and GCG mice to 400 pA current injection. Peak frequency (Fp) was obtained from the initial firing segment, and steady-state frequency (Fs) was obtained at the end of the spike train. (C, D) Plots of spike frequency adaptation (SFA), Fp divided by Fs, in WT and GCG mice at baseline and during bath application of 5 µM carbachol. (E) Comparison of normalized SFA between WT and GCG mice. Data are presented as mean ± SEM (WT, n=22 cells/18 mice; GCG, n=21 cells/15 mice). Statistical significance was determined using a two-way repeated-measures ANOVA with Tukey’s post-hoc test. *P < 0.05, **P < 0.01, ***P < 0.001. BL, baseline, CCh, carbachol.

**Supplementary Figure 4: A higher carbachol concentration raises the depolarization block threshold in GCG mice.**

**Supplementary Figure 4:** **A higher carbachol concentration raises the depolarization block threshold in GCG mice.**

(A,B) Carbachol (20 µM) enhances evoked firing activity in both WT and GCG CA1Ps.
(C, D) Plots of spike frequency adaptation (SFA) in response to depolarizing current injections in WT and GCG mice. (E) Histogram of current thresholds for depolarization block in WT and GCG mice at baseline and during CCh exposure. (F, H, J) Quantification of rheobase (H), input resistance (J), and resting membrane potential (L) at baseline and during 20 µM CCh exposure.
(G, I, K) Normalized carbachol response for rheobase (I), input resistance (K), and resting membrane potential (M). Data are presented as mean ± SEM (WT, n=11 cells/7 mice; GCG, n=12 cells/8 mice). Statistical significance was determined using a two-way repeated-measures ANOVA with Tukey’s post-hoc test for firing frequency, SFA, rheobase, input resistance, and resting membrane potential. A mixed-effects model with Fisher’s LSD post hoc analysis was used for depolarization block current thresholds. A Student’s unpaired t-test was used for normalized data. *P < 0.05, **P < 0.01, ***P < 0.001. BL, baseline, CCh, carbachol.

**Supplementary Figure 5: Current at depolarization block during 10 μM Nicotine and 10 μM Pilocarpine.**

**Supplementary Figure 5: Current at depolarization block during 10 μM Nicotine and 10 μM Pilocarpine.**
(A) Histogram of depolarization block current thresholds for cells exposed to 10 µM nicotine (WT: n=12 cells/6 mice; GCG: n=6 cells/3 mice). (B) Histogram of depolarization block current thresholds for all cells exposed to 10 µM pilocarpine (WT: n=11 cells/10 mice; GCG: n=11 cells/7 mice). (C) Histogram of depolarization block current thresholds for cells that exhibited increased excitability in response to 10 µM pilocarpine (WT: n=8 cells/ mice; GCG: n=8 cells/7 mice). (D) Histogram of depolarization block current thresholds for cells that exhibited decreased excitability in response to 10 µM pilocarpine (WT: n=3 cells/3 mice; GCG: n=4 cells/3 mice). Data are presented as mean ± SEM. A mixed-effects model with Fisher’s LSD post hoc analysis was used to assess depolarization block current thresholds. *P < 0.05, **P < 0.01, ***P < 0.001.

**Supplementary Figure 6:** **Cholinergic receptor expression is altered in the dentate gyrus of GCG mice.**

**Supplementary Figure 6:** **Cholinergic receptor expression is altered in the dentate gyrus of GCG mice.**

(A) Quantification of western blot data showing cholinergic receptor expression. Target protein levels were normalized to total protein. (B) Representative western blot images of muscarinic receptors (M1, M2, M4) and nicotinic acetylcholine receptor subtype (α3, α4, α7, β2, β4). Data are presented as mean ± SEM (WT, n=6 or 12; GCG, n=6). Statistical significance was determined using unpaired t-tests. *Q < 0.05, **Q < 0.01, ***Q < 0.001. WB= whole brain.
