# Supplementary material for: Cholinergic modulation of hippocampal CA1 pyramidal cell excitability in ArxGCG+7 mice
Source: Exp Neurol. Author manuscript; Available in PMC 2026 May 7. (PMC13150844; doi:10.1016/j.expneurol.2025.115591)
Supplement: Supplementary Material 2 [file NIHMS2157859-supplement-Supplementary_Material_2.docx]

**Full Western Blots**

**For Figure 8**

**For Supplementary Figure 6**
